# Synthesizer: Chemistry‐Aware Machine Learning for Precision Control of Nanocrystal Growth

**DOI:** 10.1002/adma.202509472

**Published:** 2025-11-05

**Authors:** Nina A. Henke, Leo Luber, Ioannis Kouroudis, Jonathan Paul, Alexander Schuhbeck, Lukas M. Rescher, Tizian Lorenzen, Veronika Mayer, Knut Müller‐Caspary, Bert Nickel, Alessio Gagliardi, Alexander S. Urban

**Affiliations:** ^1^ Nanospectroscopy Group and Center for NanoScience (CeNS) Nanoinstitute Munich Department of Physics Ludwig‐Maximilians‐Universität (LMU) München 80539 Munich Germany; ^2^ Chair of Simulation of Nanosystems for Energy Conversion, Department of Electrical Engineering TUM School of Computation, Information, Technology Atomistic Modeling Center (AMC) Munich Data Science Institute (MDSI) Technische Universität München (TUM) 85748 Garching Germany; ^3^ Soft Condensed Matter Group and Center for NanoScience (CeNS) Department of Physics Ludwig‐Maximilians‐Universität (LMU) München 80539 Munich Germany; ^4^ Department of Chemistry and Center for NanoScience (CeNS) Ludwig‐Maximilians‐Universität (LMU) München 81377 Munich Germany

**Keywords:** antisolvent engineering, Gaussian processes, machine learning, perovskite nanocrystals, photoluminescence optimization, synthesis design

## Abstract

Precise and reproducible control over nanocrystal synthesis is essential for tailoring optical properties, yet remains a long‐standing challenge in halide perovskites. A broadly adoptable machine learning–guided framework, the Synthesizer, is introduced that combines Gaussian Process regression and Bayesian optimization with chemistry‐aware molecular encodings and systematic feature engineering. Rather than new algorithms, the advance lies in translating interpretable machine learning tools into a practical, benchtop platform for nanocrystal optimization under ambient conditions. Using CsPbBr_3_ as a model system, nm‐level precision in photoluminescence peak tuning (430 nm to 520 nm) is achieved, along with benchmark narrow linewidths down to 70 meV via lateral confinement control, and robust photoluminescence quantum yield optimization linked to surface trap density. Mapping the two‐dimensional parameter space (Cs/PbBr_2_ and antisolvent/PbBr_2_ ratios) across multiple antisolvents enables predictive optimization and identifies the antisolvent/PbBr_2_ ratio as a previously underappreciated mechanistic parameter, offering a quantitative basis for antisolvent‐accelerated nanocrystal growth. Transfer tests across distinct chemical spaces, including alcohols and cyclopentanone, confirm generalizability to unseen molecules, while application to CsPbI_3_ demonstrates extension to new material systems. These results establish an adoption‐ready platform for data‐efficient, uncertainty‐aware synthesis design, providing reproducible pathways to accelerate materials discovery beyond halide perovskites.

## Graphical abstract

A new, data‐efficient approach to CsPbBr_3_ nanocrystal optimization combines chemical and physical insights with a Gaussian Process‐based machine learning algorithm. Implementation of the “Synthesizer” enables nm‐precise tuning of the emission wavelength while reducing experimental load in optimizing peak narrowness and photoluminescence quantum yield. The method demonstrates adaptability and transferability to other systems, underscoring its potential for broader material optimization.

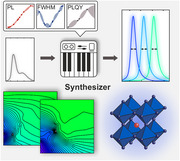

## 1 Introduction

The transition to renewable energies, reduced energy consumption, and greater sustainability are ever more critical issues in light of increasing global temperatures and frequent severe weather events. Nanomaterials, with unique size‐dependent properties, already play an important role in this effort, for example, by improving the efficiency and durability of solar cells, batteries, and fuel cells through improved charge transport, light absorption, and catalytic activity.^[^ ^1^, ^2^, ^3^, ^4^ ^]^ Lead halide perovskite nanocrystals have attracted significant interest for photocatalysis and optoelectronic applications, including photovoltaics and lighting, due to a wealth of properties crucial for commercialization.^[^ ^5^, ^6^, ^7^, ^8^, ^9^, ^10^ ^]^ This auspicious material class demonstrates color tunability in the entire visible range by adjusting the composition, size, or shape, achieves high photoluminescence quantum yields (PLQY) and allows facile, scalable synthesis and processing methods.^[^ ^11^, ^12^, ^13^, ^14^ ^]^ Moreover, specifically tailoring the geometry to produce 2D nanoplatelets (NPLs) leads to even narrower emission spectra and directional emission, benefitting light outcoupling and boosting external quantum efficiencies of light‐emitting diodes (LEDs).^[^ ^15^, ^16^, ^17^, ^18^, ^19^ ^]^ However, achieving narrow, bright, and wavelength‐tunable emission remains experimentally challenging—particularly for the quantum‐confined NPLs—due to their sensitivity to reaction conditions, surface defects, and environmental degradation.^[^ ^20^, ^21^, ^22^, ^23^ ^]^ Complicating the optimization is the fact that perovskite nanocrystal growth is still not sufficiently understood in general, and insights into the reaction mechanism are difficult to obtain due to the rapid reaction kinetics.^[^ ^24^, ^25^, ^26^ ^]^

Although systematic correlations between precursors, synthesis parameters, and the eventual size, shape, crystal structure, and optical properties of resulting nanocrystal ensembles have been established, the absence of mechanistic understanding impairs the fabrication of tailored perovskite nanocrystals. Conventional approaches to material optimization still rely on empirical synthesis strategies and often involve time‐consuming synthesis parameter screenings, varying one parameter at a time in a complex parameter space.^[^ ^27^ ^]^ Machine learning has become an increasingly powerful tool for accelerating materials discovery and synthesis optimization, including in halide perovskites.^[^ ^27^, ^28^, ^29^ ^]^ Classical algorithms, neural networks, and random forests have demonstrated good predictive performance, but typically require large datasets and lack intrinsic uncertainty quantification, limiting their effectiveness in data‐scarce experimental settings.^[^ ^30^, ^31^ ^]^ In contrast, Gaussian Processes provide full uncertainty awareness and are particularly well‐suited to the low‐data regime.

Here, Bayesian optimization has been shown to be the most robust choice in process optimization, as has been showcased in early applications and metamaterials.^[^ ^32^, ^33^, ^34^ ^]^ Machine learning has also been applied to nanocrystal synthesis in several forms, including hardware‐intensive robotic platforms,^[^ ^35^, ^36^ ^]^ microfluidic flow systems,^[^ ^37^, ^38^ ^]^ and low‐data GP/BO frameworks,^[^ ^39^ ^]^ as well as more recent multi‐objective optimization of colloidal QDs.^[^ ^40^ ^]^ While robotic and flow‐based approaches achieve impressive autonomy, they require specialized infrastructure, and prior low‐data studies typically optimize single objectives such as emission peak position. In this work, we introduce a lightweight, benchtop framework that jointly optimizes peak wavelength, linewidth, and PLQY under ambient conditions and demonstrates adaptability to a new perovskite system. In contrast to autonomous robotic and microfluidic platforms, our framework is deliberately benchtop‐compatible and infrastructure‐light, enabling rapid adoption in standard labs. While self‐driving systems emphasize throughput, “Synthesizer” emphasizes data efficiency, uncertainty‐aware decision making, and chemical interpretability, delivering nm‐precise control of color with narrow linewidths and strong PLQY under ambient conditions. This positions Synthesizer as a complement to hardware‐intensive approaches by providing a broadly accessible pathway to ML‐guided synthesis optimization. Such models form the basis of data‐efficient synthesis design frameworks, including our previously reported algorithm for thickness‐controlled CsPbBr_3_ NPLs.^[^ ^39^, ^41^, ^42^, ^43^, ^44^ ^]^

This work builds on our previous efforts to apply machine learning to halide perovskite nanocrystal synthesis. In our former study, we combined multiple machine learning models with Bayesian optimization to improve spectral purity in CsPbBr_3_ NPLs, primarily targeting peak symmetry and narrowness using precursor ratios as input parameters. While that approach enabled data‐efficient optimization within a constrained synthetic space, it lacked structural generality and molecular transferability. Rather than proposing new ML algorithms, we focus on building a synthesis‐guiding platform that bridges chemical complexity with data‐efficient predictive modeling. Accordingly, our current framework, the Synthesizer, significantly expands both the parameter space and predictive scope by incorporating molecular descriptors via a geometric encoding of antisolvents, allowing synthesis design based on molecular identity, not just composition. We also introduce predictive modeling for multiple optical properties, demonstrate lateral size control, and validate transferability to new systems. This broader, more versatile platform enables guided exploration across synthesis targets and material systems, addressing limitations of prior approaches while maintaining data efficiency. We emphasize that the novelty of this work lies not in proposing a new algorithm, but in creating a generalizable synthesis platform that integrates chemically meaningful encodings with uncertainty‐aware optimization. This integration enables reproducible, precise synthesis control under ambient conditions, providing a transferable framework for data‐driven materials discovery.

In our new platform, we optimize specified optical properties of a chosen model system, namely CsPbBr_3_ nanocrystals synthesized under ambient conditions. Specifically, we evaluate the impact of precursor ratios and the use of different antisolvents on the photoluminescence (PL) peak wavelength, peak narrowness, and PLQY of the resulting colloidal nanocrystal solutions. In optoelectronic applications, the PL peak wavelength, spectral width or full width at half maximum (FWHM), and PLQY are the defining parameters for performance. Together, they determine the color, color purity, and brightness of nanocrystal emission—properties that are crucial for achieving the desired spectral response in devices such as LEDs, lasers, and displays. However, optimizing all three simultaneously remains challenging, especially in highly sensitive systems like halide perovskites. Our goal is to develop a synthesis optimization framework that enables precise, data‐efficient tuning of these properties, empowering researchers to systematically design nanocrystals with targeted emission characteristics that are reproducible and customizable. In conjunction with chemistry‐informed data augmentation and experimental sampling in the reduced two‐dimensional parameter space, given by the molar Cs/PbBr_2_ precursor ratio and the molar antisolvent/PbBr_2_ ratio, we achieve nanometer‐level precision in predicting PL peak wavelength, thereby enabling fine control over emission color between 430‐520 nm, a key requirement for applications in displays, lighting, and quantum photonics. Our approach leverages an encoding of the antisolvent geometry as well as an antisolvent‐independent baseline to extrapolate the parameter space to unknown antisolvents, thereby drastically reducing the number of syntheses required for accurate predictions. A systematic selection of antisolvents results in a reduced peak narrowness and excellent size homogeneity of blue‐green emitting CsPbBr_3_ nanocrystals. Moreover, the first‐ever report of controlled lateral size tuning in the quantum‐confined regime yields some of the narrowest PL profiles reported for blue‐emitting perovskite NPLs. We find that the PLQY of CsPbBr_3_ nanocrystals is antisolvent independent and instead shows a strong correlation with the nanocrystal surface‐to‐volume ratio, providing statistical evidence for the established correlation between surface defects and PLQY in lead halide perovskite materials. Lastly, we highlight how this approach to synthesis optimization can be extended to include additional synthesis parameters or applied to related material systems, such as CsPbI_3_.

## 2 Results and Discussion

**Figure 1:**
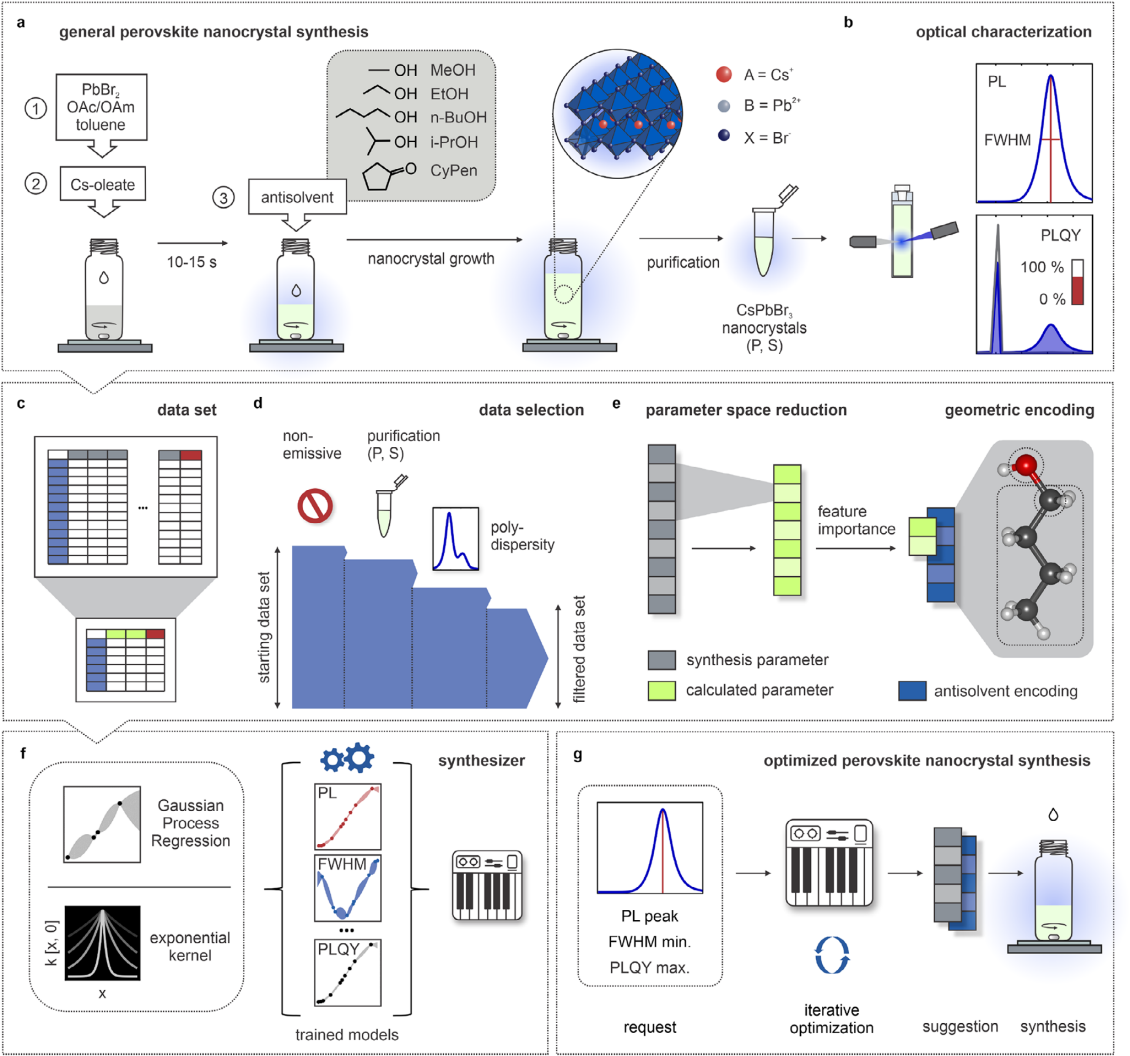
Synthesis, optical characterization, and machine‐learning workflow. a) Synthesis of CsPbBr_3_ perovskite nanocrystals by ligand‐assisted spontaneous crystallization in nonpolar solvents. Mixing of the PbBr_2_ and Cs‐oleate precursor is followed by the addition of antisolvent and results in the growth and precipitation of perovskite nanocrystals. b) The nanocrystal product in the precipitate (P) and supernatant (S) is characterized by steady‐state optical spectroscopy. PL peak wavelength and FWHM are extracted from PL spectra, and the absolute PLQY of each colloidal sample is determined. c) The initial dataset is reduced in dimension and size through a two‐step selection process. d) Data that does not meet predefined criteria (emissive product, low polydispersity, P/S‐type samples) are excluded, and e) the feature space consisting of all available synthesis parameters is reduced through calculation of relevant quantities and feature importance analysis. Characteristic antisolvent information is included by appending a 5‐dimensional encoding of the molecule geometry. f) The reduced dataset is used to train Gaussian Process Regression models with an exponential kernel on a selection of relevant targets (PL peak wavelength, FWHM, PLQY). g) Workflow of the Synthesizer optimization; an initial request contains the requested PL peak wavelength and additional demands, such as low FWHM or high PLQY that fully define the objective function of the following iterative optimization. Ideal synthesis parameters for the particular request are found and returned in the appropriate units for synthesis.

All‐inorganic CsPbBr_3_ nanocrystals were synthesized in ambient conditions, following a facile, scalable approach of ligand‐assisted spontaneous crystallization in nonpolar solvents (**Figure** **1**a). A precursor containing PbBr_2_, oleylamine (OAm), and oleic acid (OAc) ligands in toluene is mixed with a Cs‐oleate precursor. After 10–15 s, an antisolvent is injected into the reaction mix, promoting perovskite nanocrystal growth and precipitation. The crude reaction mixture is then purified by centrifugation, and CsPbBr_3_ nanocrystals are redispersed in n‐hexane for subsequent analysis. Both nanocrystals in the precipitate (P) and supernatant (S) are characterized and used for analysis. The optical properties of pristine perovskite nanocrystal products were probed immediately after synthesis by measuring the PL spectrum and PLQY of the colloidal solutions (Figure 1b). Then, the PL peak wavelength and full width at half‐maximum (FWHM) are extracted from the PL spectra. We deliberately based our machine learning optimization framework on PL properties rather than absorption properties due to the direct relevance of PL peak wavelength, FWHM, and PLQY to emissive performance in optoelectronic applications.^[^ ^45^, ^46^ ^]^ Previous studies established that strongly quantum‐confined perovskite nanocrystals can be obtained by reducing the precursor ratio Cs/PbBr_2_ in the presented room‐temperature synthesis. The specific ratio allows incremental size and color tunability in the spectral range between 430 and 510 nm.^[^ ^15^, ^16^, ^39^ ^]^ Initially believed to only be necessary to induce crystallization, the selection of antisolvent also strongly influences nanocrystal growth, whereby the shape and size of the nanocrystals CsPbBr_3_ are primarily determined by the antisolvent polarity, dipole moment, and Hansen solubility parameter.^[^ ^26^, ^47^ ^]^ Our previous work explored the underlying nanocrystal formation mechanism in the given synthesis approach. We showed that mixing of Cs‐oleate and PbBr_2_ precursors results in the formation of small crystalline intermediate CsPbBr_3_ nanocrystals.^[^ ^26^ ^]^ The effect of subsequent antisolvent injection into the reaction mixture, containing said intermediates, is two‐fold: First, the polar antisolvent headgroups can interact with weakly bound OAm^+^ ligands via hydrogen‐bonding, thereby exposing the surface of intermediate CsPbBr_3_ nanocrystals. Second, adding polar antisolvents to the nonpolar reaction mixture in toluene reduces the precursor micelle stability and increases the precursor monomer solubility, promoting a controlled release from micellar precursor reservoirs, similar to the diffusion‐mediated synthesis reported recently.^[^ ^25^, ^48^ ^]^ In combination, this enables the attachment of precursor material to intermediate CsPbBr_3_ nanocrystals and their growth to larger nanocrystals. However, the quantitative correlation between antisolvent injection and nanocrystal growth and the limits of tunability have not yet been explored. Here, we select methanol (MeOH), ethanol (EtOH), isopropanol (i‐PrOH), butanol (n‐BuOH), and cyclopentanone (CyPen) as suitable antisolvent candidates based on availability and findings from previous work.^[^ ^26^ ^]^ As the influence of other synthesis parameters like reaction temperature, ligand concentration, ligand ratio, and ligand chain length have already been investigated elsewhere,^[^ ^49^, ^50^ ^]^ we kept these constant. Constraints from experimental limitations were introduced to define the accessible parameter space, including minimum and maximum precursor concentrations and precursor and antisolvent volumes (Table S1, Supporting Information). Most importantly, the molar Cs/PbBr_2_ ratio was limited to 0.0–1.0 to avoid the formation of non‐emissive Cs_4_PbBr_6_ or CsPb_2_Br_5_ phases.^[^ ^50^, ^51^, ^52^ ^]^

### 2.1 Synthesizer

The experimental workflow described above produces a data set containing synthesis parameters (independent variables) such as precursor volumes and concentrations, antisolvent type and volume, as well as extracted values for the optimization targets (dependent variables), such as PL peak wavelength, FWHM, and PLQY values. The initial dataset, comprising 79 samples from prior syntheses conducted in our laboratory, provided a representative foundation for initializing the Bayesian optimization framework and was subsequently expanded during the optimization process. For clarity, perovskite nanocrystal products were categorized according to their PL peak wavelength, that is, their emission color (Table S2, Supporting Information).

The available data set was then substantially reduced in size and dimension through a multi‐step selection process (Figure 1c). For this, parameter combinations from syntheses that do not produce emissive CsPbBr_3_ nanocrystals or yield polydisperse samples with multiple PL peaks were omitted. Additionally, data from nanocrystals in the precipitate (P) or supernatant can also be selectively excluded, depending on the specific synthesis request and overall size of the available data set (Figure 1d). Second, the dimensionality of the explored parameter space consisting of all available synthesis parameters is reduced through the calculation of relevant combined quantities (Figure 1e) and feature importance analysis (Figure S1, Supporting Information). This removes uncorrelated parameters and ultimately yields two expressive molar ratios, Cs/PbBr_2_ and antisolvent (as)/PbBr_2_, which adequately define perovskite nanocrystal growth in the presented synthesis. Third, the type of antisolvent is included by appending a 5D encoding of the molecule geometry, including its functional group as a one‐hot encoding as well as the alkyl chain length, and the alkyl chain structure. We deliberately chose this low‐dimensional geometric encoding over more complex molecular fingerprints or physicochemical descriptors, because the limited number of available antisolvents renders high‐dimensional encodings prone to overfitting. Moreover, using specific molecular properties (e.g., Hansen parameters, dipole moment) would introduce bias toward assumed interaction mechanisms, whereas our geometry‐based encoding remains agnostic and flexible, while still capturing all relevant structural features. This encoding acts as a robust similarity measure between the respective antisolvent molecules. As such, it represents a low‐dimensional identifier capturing all relevant features of the antisolvents used in this work yet can easily be extended to more complex molecular geometries. For example, other functional groups might be included by simply extending the length of the corresponding one‐hot encoding. Since the selection of suitable antisolvents does not include arbitrary levels of complexity, a more general and therefore high‐dimensional identifier is not required. Finally, a set of Gaussian Process regressors is trained on the reduced data set, with each one mapping the parameter space onto a single target (PL peak wavelength, FWHM, PLQY), thereby modeling the underlying chemical and physical processes. An exponential kernel was chosen to focus on short‐distance dependencies, accounting for strongly localized features in the synthesis data, like plateaus and high gradients (Figure 1f). An antisolvent‐independent baseline data set was introduced to constrain the model at the parameter space boundaries and to avoid overfitting in areas with fewer data points. The trained predictors, in combination with the data pre‐processing and the optimization algorithm, make up the Synthesizer. Based on the input synthesis parameters, this framework can accurately predict the peak wavelength and FWHM of the PL spectrum as well as estimate the PLQY of resulting colloidal CsPbBr_3_ nanocrystals. The process of a Synthesizer‐guided synthesis is shown in Figure 1g. An initial request comprises a desired emission wavelength and additional weighted demands like minimized peak narrowness (FWHM) or maximized emission efficiency (PLQY). These, together with general constraints on the synthesis parameters, define the objective function for optimization. Points in the parameter space are sampled within a predefined maximum number of iterations. The best set of parameters is then denormalized and transformed to the initial parameters, yielding a final, optimized synthesis suggestion. The optical characterization of the resulting perovskite nanocrystal product can then be added to the data set to create a feedback loop and adjust the hyperparameters of the optimization. At submission of this manuscript, the Synthesizer operates on a data set including synthesis parameters and optical characterization results of 287 CsPbBr_3_ nanocrystal samples (Table S3, Supporting Information).

### 2.2 Accurate Prediction of Photoluminescence Peak Wavelength

**Figure 2:**
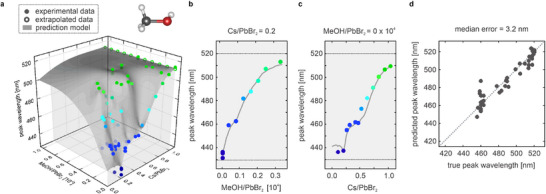
Prediction of PL peak wavelength from 2D parameter space. a) 2D parameter space defined by the molar Cs/PbBr_2_ and MeOH/PbBr_2_ ratios with corresponding true PL peak wavelengths (colored dots) and the fully trained regression model (dark grey surface) for reference. Antisolvent independent synthesis data points at as/PbBr_2_ = 0 and extrapolated data points at Cs/PbBr_2_ = 1 set a frame at the parameter space boundaries to constrain the model in sparse‐data regimes. Systematic synthesis screenings at constant b) Cs/PbBr_2_ ratio and c) constant MeOH/PbBr_2_ ratio demonstrate excellent control over PL peak wavelength in the range between 430 and 520 nm. d) The Leave‐One‐Out (LOO) regression plot compares the true and predicted PL peak wavelength, achieving a high precision of ±3 nm (median error) and ±5 nm (mean error) that is within the emission range of individual CsPbBr_3_ nanocrystal products.

Having established the procedure for nanocrystal synthesis, characterization, data pre‐processing, and optimization, we analyze the effects of individual synthesis parameters on the optical properties of the perovskite nanocrystal products. Our previous study revealed the importance of the antisolvent, which we now investigate in more detail.^[^ ^26^ ^]^ The feature reduction of the Synthesizer produces two new parameters, the molar ratios Cs/PbBr_2_ and as/PbBr_2_, resulting in a two‐dimensional parameter space. We explored the parameter space for different antisolvents, as shown for the samples synthesized with MeOH in **Figure** **2**a. The obtained nanocrystal dispersions exhibit PL peaks centered between 430 and 520 nm, with the emission wavelength redshifting as either one or both ratios increase. While the Cs/PbBr_2_ ratio is a known synthesis parameter for controlling the nanocrystal dimensions and PL peak wavelength of lead halide perovskites,^[^ ^53^ ^]^ the herein established molar as/PbBr_2_ ratio has an equally strong effect on the PL peak wavelength (Figure S1, Supporting Information), as confirmed by the results from two series of syntheses depicted in Figure 2b,c. In combination, these two ratios allow for precise control of CsPbBr_3_ nanocrystal emission between 430 and 520 nm. For MeOH as the antisolvent, deep‐blue emission below 450 nm is only accessible within the no‐antisolvent limit. The characteristic shape of the PL peak wavelength mapping depicted in Figure 2a is accurately reproduced by the Gaussian Process Regression. It captures all relevant features in the data, such as the small plateaus found for deep‐blue and pure‐blue emission between 430 and 440 nm, and 455 and 465 nm, respectively. Plateaus correspond to areas where the nanocrystal product hardly changes as the synthetic parameters vary. Here, they mark distinct populations of 2 monolayer (ML) and 3ML thick NPLs, whereby one ML refers to a single layer of corner‐sharing PbBr_6_ octahedra.^[^ ^16^, ^26^ ^]^ Employing other alcohols as antisolvents (EtOH, i‐PrOH, n‐BuOH), the plateau of blue‐emitting 3ML NPLs is more strongly pronounced, and the threshold of minimum as/PbBr_2_ ratio required for obtaining nanocrystal products with redshifted emission is increased (Figures S2 and S3, Supporting Information). We attribute this to a lower antisolvent strength of alcohols with longer or branched alkyl chain lengths.^[^ ^47^, ^54^, ^55^ ^]^

To constrain the model in sparse data regimes and set a frame at the parameter space boundaries, antisolvent independent synthesis data points at as/PbBr_2_ = 0, Cs/PbBr_2_ = 0–1, and extrapolated data points at Cs/PbBr_2_ = 1, as/PbBr_2_ = 0–1 are added (Figure S4, Supporting Information). Both implementations are based on the assumption that antisolvent addition only results in samples with identical or red‐shifted emission compared to a synthesis for a set Cs/PbBr_2_ ratio without antisolvent. This holds for all experimental data, and these additional data points constitute excellent continuations of the results. We note that a high sensitivity for small local features is generally prone to overfitting, in which case experimental noise and outliers can be mistaken for local features and negatively impact the regression performance. To test for overfitting, Leave‐One‐Out (LOO) accuracies are determined and monitored for all trained models. Based on the parameter space explored in syntheses, the Synthesizer regression allows for a precise mapping of unknown points in the parameter space to the PL peak wavelength with ±3 nm accuracy for MeOH as antisolvent (Figure 2d). For EtOH, i‐PrOH, n‐BuOH, and CyPen antisolvent, equally high prediction accuracies between ±1 nm and ±4 nm (median error) or between ±3 nm and ±5 nm (mean error) are achieved (Figures S5 and S6, Supporting Information). These results demonstrate the effectiveness of the feature selection and show that these two parameters are sufficient to accurately predict the PL peak wavelength of CsPbBr_3_ nanocrystals prepared by ligand‐assisted spontaneous crystallization. Beyond optimization, these results provide mechanistic insight into the synthesis process. The Cs/PbBr_2_ ratio has long been recognized as a determinant of emission energy, whereas the as/PbBr_2_ ratio emerges here as a previously underappreciated but equally critical tuning parameter. Mapping PL peak positions across this two‐dimensional space for different antisolvents offers a quantifiable measure of the antisolvent‐induced acceleration of nanocrystal growth, establishing a basis for future mechanistic models.

### 2.3 Optimized Photoluminescence Peak Narrowness

**Figure 3:**
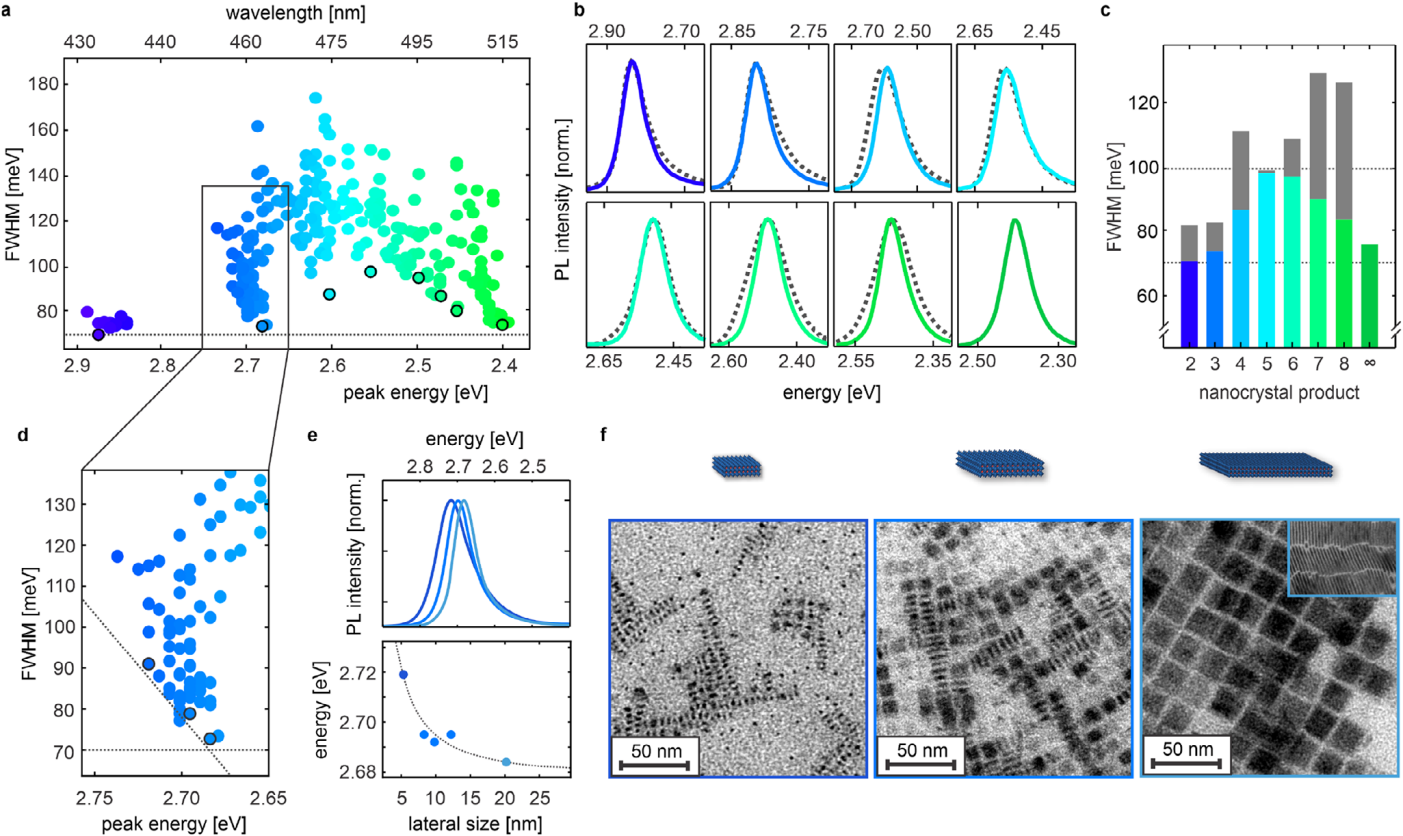
Optimization of FWHM in CsPbBr_3_ nanocrystals. a) Peak narrowness, described by FWHM, of emissive perovskite nanocrystals synthesized in this work. Optimized samples for each nanocrystal product are circled in black. b) Comparison of optimized PL spectra from this work (color) with optimized PL spectra from a similar synthesis approach without antisolvent variation (dashed grey lines). CsPbBr_3_ nanocrystals with green emission were only achieved through antisolvent variation. c) FWHM of PL spectra shown in (b), a notable improvement is observed for all nanocrystal products, resulting in nanocrystals with extremely narrow (FWHM 70–100 meV) and color‐pure emission in the entire blue to green spectral range. d) Enlarged section of the pure blue emission range. The FWHM decreases linearly with the PL peak energy toward an apparent material‐specific minimum at 70 meV, indicated by dashed grey lines. e) PL spectra of 3ML CsPbBr_3_ NPLs, corresponding to the three data points highlighted in d). The emission energy decreases slightly with increasing lateral size, which is adequately described by an inverse power law fit. f) Transmission electron microscopy (TEM) images of nanocrystal samples in **e**, showing CsPbBr_3_ NPLs with 3ML thickness and varying lateral sizes of (5.3 ± 0.9) nm, (12.2 ± 1.3) nm and (20.2 ± 1.4) nm.

With the ability to synthesize nanocrystals with desired emission wavelengths, we now focus on optimizing the color purity of perovskite nanocrystals by analyzing the PL peak narrowness, defined by the FWHM. A low FWHM generally corresponds to a high nanocrystal size uniformity and high emission color purity. **Figure** **3**a shows a summary of FWHM values for all emissive samples with respect to their PL peak energy. The synthesized CsPbBr_3_ nanocrystals possess FWHM values of 70‐180 meV, and data points cluster around emission wavelengths ascribed to NPLs of increasing ML thickness.^[^ ^16^, ^56^, ^57^ ^]^ We extracted the narrowest emission peak for each nanocrystal product (circled data points), and compared the spectra with previously reported best results from a similar synthesis approach (Figure 3b).^[^ ^39^ ^]^ The specific variation of antisolvent type and as/PbBr_2_ ratio combined with a variation of Cs/PbBr_2_ ratio in the synthesis results in significantly narrower emission profiles for all perovskite nanocrystal products. This is evident from a comparison of the FWHM values for each product (Figure 3c). The FWHM was improved by up to 50 meV, especially for light‐blue (nanocrystal product 4) and greenish emission (nanocrystal products 7–8). Furthermore, the variation of antisolvent type allows for the preparation of weakly confined nanocrystals with narrow green emission and good size homogeneity, which was impossible in the previous synthesis approach.^[^ ^39^ ^]^

Overall, the narrowest PL spectra were achieved for the highest and lowest PL peak energies, whereas PL spectra centered between 480‐500 nm exhibit higher average FWHM values. Interestingly, within a specific energy range, for example, the pure blue emission range (nanocrystal product 3), the minimal FWHM shows a direct linear anticorrelation with decreasing PL peak energy (Figure 3d,e). To understand this behavior, we employed transmission electron microscopy (TEM) on different nanocrystal samples emitting in this region (Figure 3f). We confirm that these are 3ML‐thick NPLs. However, their lateral sizes vary from (5.3 ± 0.9) nm to (20.2 ± 1.4) nm (Figure S7, Supporting Information). Larger 3ML NPLs emit at slightly longer wavelengths and possess narrower PL spectra. This could be due to their lateral size significantly exceeding the exciton Bohr radius of CsPbBr_3_.^[^ ^5^, ^53^ ^]^ While the NPLs are monodisperse in thickness, they exhibit a minor polydispersity in the lateral dimension, significantly impacting the inhomogeneous broadening of the PL spectrum of laterally smaller NPLs. Alternatively, the ligands might have a pronounced effect on the crystal structure and thereby the energetic band structure as seen for small halide perovskite nanocubes.^[^ ^58^ ^]^ Regardless of the origin, the smaller the NPLs are laterally, the further the PL spectrum is shifted to higher energy. Therefore, we can exploit the PL peak prediction of the Synthesizer to achieve extremely narrow pure blue emission. We find that a straightforward prediction of the FWHM from a given set of synthesis parameters is possible with the Synthesizer when trained throughout the entire wavelength range, and an accurate prediction of the PL peak wavelength is guaranteed (Figure S8, Supporting Information). This allows for a direct FWHM optimization, either via a synthesis suggestion for the respective emission range of each nanocrystal product or by a targeted prediction aimed at the upper wavelength limit. We find that slightly increasing the Cs/PbBr_2_ ratio and decreasing the overall concentration in the synthesis yields 3ML NPLs with large lateral sizes that exhibit extremely narrow pure blue spectra with FWHM = 74 meV. To underline the generality of the approach, we also demonstrate the lateral size tuning for the FWHM optimization of 2ML NPLs (nanocrystal product 2, Figure S9, Supporting Information) and 4ML NPLs (nanocrystal product 4, Figure 10, Supporting Information), resulting in the narrowest blue perovskite emission spectra ever reported.^[^ ^59^, ^60^ ^]^

Similarly, the size and shape of nanocrystals emitting in the spectral range between 480 and 510 nm (nanocrystal products 5‐8) were also analyzed with TEM. Here, images show cuboid particles with excellent size uniformity and dimensions of (4.3 ± 0.5) nm, (5.4 ± 0.5) nm, (6.3 ± 0.7) nm, and (8.7 ± 0.8) nm, respectively (Figure S14, Supporting Information). Since TEM imaging can be ambiguous in distinguishing platelets and cubes, depending on the observed nanocrystal orientation, we also confirm the nanocrystal shape and size with small‐angle X‐ray scattering (SAXS) in solution (Figure S15, Supporting Information). We note that SAXS indeed hints at a minor anisotropy of CsPbBr_3_ nanocrystal samples with sky‐blue emission (nanocrystal product 5) that is undiscernible in TEM images. Based on these results, the slightly increased FWHM is attributed to the difference in the nanocrystal shape. This is due to inhomogeneous broadening of the PL profiles being affected by size polydispersity in three dimensions for the nanocubes. Compared to similar, isotropic CsPbBr_3_ nanocrystals, the PL spectra of optimized CsPbBr_3_ nanocrystals with blue‐green and green emission presented in this work are also among the narrowest ever reported.^[^ ^61^, ^62^, ^63^ ^]^ Interestingly, there appears to be a material‐specific minimal linewidth broadening at room temperature, likely of homogeneous origin, such as exciton‐phonon‐coupling.^[^ ^64^ ^]^ Overall, the experimental data collected in this project enable the optimization of individual nanocrystal products due to an understanding of the synthesis and resulting nanocrystal morphology. Obtaining monodisperse samples of large anisotropic NPLs with 5‐8ML thickness should further reduce FWHM values throughout the blue‐green spectral range. However, this will require a variation of additional parameters in the synthesis protocol, i.e., the reaction temperature, the ligand system, or the usage of still untested antisolvents to promote anisotropic growth.^[^ ^57^, ^65^ ^]^

### 2.4 Optimization Through Antisolvent Choice

**Figure 4:**
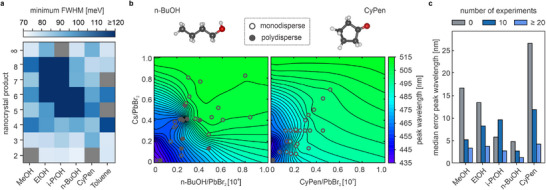
FWHM optimization through antisolvent selection. a) A heatmap of minimal FWHM values obtained from syntheses with different antisolvents points to inherent differences in the nanocrystal growth when introducing antisolvents with different molecular geometries. Grey fields indicate no data for the respective product/antisolvent combination. b) Representative contour plots for nanocrystal syntheses with n‐BuOH and CyPen reveal the distinct behavior of the PL peak wavelength. In general, regions of steep gradients typically correlate with polydisperse products, as seen for samples with PL peak wavelengths between 480 and 500 nm when synthesized with n‐BuOH. In contrast, the contour plot of CyPen exhibits a shallower gradient in the same spectral region, resulting in monodisperse samples with benchmark FWHM values < 100 meV. c) Basic molecular transfer gives a rough estimation of the PL peak wavelength for unknown antisolvents (0 experiments) with an accuracy strongly dependent on overall geometric similarity. However, performing 10–20 syntheses is sufficient to reach a prediction accuracy of <±5 nm (median error) or <±6 nm (mean error).

The data in Figure 3 includes syntheses with different antisolvents, and the question remains how the selection of antisolvent affects the PL peak narrowness of the resulting nanocrystals. As shown in **Figure** **4**a, the minimum FWHM achieved for different nanocrystal products varies significantly from one antisolvent to another. Alcohols and toluene yield ensembles of 2ML NPLs, 3ML NPLs, and weakly confined nanocrystals with narrow FWHM, while ensembles of other nanocrystal products only achieve a FWHM of 100–120 meV or above. Very broad PL spectra are likely caused by polydisperse ensembles containing multiple different perovskite nanocrystal products. In contrast, syntheses with CyPen produce CsPbBr_3_ nanocrystal ensembles with a narrow PL peak at any given wavelength between 470 and 510 nm. To analyze this in detail, we derive a two‐dimensional map of the parameter space spanned by the Cs/PbBr_2_ and as/PbBr_2_ ratios that indicates the PL peak wavelength for each antisolvent. Notably, the PL peak wavelength mappings correlate strongly with the previous results, as shown in Figure 4b for n‐BuOH and CyPen.

In steep‐gradient regions, such as between 480 and 500 nm, even a minute variation of the Cs/PbBr_2_ or as/PbBr_2_ ratio results in a significant shift of the resulting PL peak, and therefore a different nanocrystal product. Consequently, isolated perovskite nanocrystal ensembles obtained in this region of the parameter space are often polydisperse, exhibiting either severely broadened or multiple PL peaks. The step function of the PL peak wavelength map of n‐BuOH is also found for EtOH and i‐PrOH, leading to equally polydisperse nanocrystal products in steep‐gradient regions between 480 and 500 nm (Figure S11, Supporting Information). In regions with a shallower gradient, a slight variation of Cs/PbBr_2_ or as/PbBr_2_ ratio yields an identical nanocrystal product. It thus enables a reproducible synthesis of uniform perovskite nanocrystal ensembles with narrow PL spectra. Estimating the map profile for unknown antisolvents is enabled by the transfer capabilities of the Synthesizer. While the antisolvent independent baseline sets constraints at the limits of the 2D parameter space, the geometric encoding contains an array of structural information about the specific molecule. This allows the model to draw information about gradients, plateaus, and step‐function‐like features of the PL peak wavelength map from geometrically similar antisolvents in the training data. These geometric similarities, in turn, translate to a similar effect on the NC growth as seen from the consistent, slightly shifted mapping of linear alcohols (Figure 4b; Figure S11a,b, Supporting Information) compared to i‐PrOH (Figure S11c, Supporting Information).

To verify the validity of this approach, we excluded all syntheses with a specific antisolvent from the data set and trained the Synthesizer on the remaining data. We then asked the Synthesizer to predict the PL peak wavelength for syntheses with the excluded antisolvent and compared the predictions with the experimental results (Figure 4c). Repeated for all tested antisolvents, the initial median error in the PL peak wavelength is around ±15 nm for MeOH and EtOH, or even as low as ±5 nm for i‐PrOH and n‐BuOH. The initial transfer error (0 syntheses) is much higher for CyPen because there are no structurally similar molecules in the data set to draw information from. However, if we incorporate experiments with the chosen antisolvent, simulating a couple of syntheses, the median error rapidly decreases to less than ±10 nm (10 syntheses), and ultimately to less than ±5 nm (⩾ 20 syntheses), regardless of the geometry. The mean transfer errors amount to similar values, as illustrated in Figure S12 (Supporting Information), and the general evolution of PL peak wavelength and FWHM prediction accuracy is included in Figure S13 (Supporting Information). Because the geometric encoding was established prior to resolving any antisolvent‐specific trends, the leave‐one‐antisolvent‐out regressions in Figure 4c constitute true transfer tests to unseen chemical spaces. The inclusion of structurally diverse molecules, such as the cyclic ketone cyclopentanone versus the linear alcohols, further underscores the robustness of this validation. We note, however, that expanding the antisolvent set to cover a broader range of molecular motifs would strengthen transferability even further, and this is a focus of our ongoing work.

While the functional group of the antisolvent is crucial for the successful synthesis of CsPbBr_3_ nanocrystals, knowledge of the geometric similarity factor is advantageous to reduce experimental efforts.^[^ ^26^ ^]^ It can be exploited for an accelerated screening of new antisolvents. Ultimately, the PL peak narrowness of a targeted nanocrystal product, i.e., a requested emission wavelength, can be optimized through the selection of the antisolvent. Our results show that nanocrystals with narrower emission profiles are obtained with antisolvents that possess a shallower emission wavelength gradient. Here, the Synthesizer can be applied to efficiently select promising antisolvents from a minimal number of syntheses or, in the case of geometrically similar molecules, without any additional data. Accordingly, 2ML, 3ML NPLs, and weakly confined nanocrystals (nanocrystal products 2,3, and ∞) are best synthesized with alcohols as antisolvents. All other remaining nanocrystal products 4‐8 are best obtained with CyPen (Table S4, Supporting Information). A comparison of relevant antisolvent properties (Table S5, Supporting Information) reveals significant differences between MeOH, EtOH, i‐PrOH, n‐BuOH, and CyPen. We hypothesize that, unlike alcohol‐based antisolvents, where substantial hydrogen‐bond donation facilitates rapid ligand desorption^[^ ^66^ ^]^ and less‐controlled, burst‐like growth of CsPbBr_3_ nanocrystals, weaker hydrogen‐bonding of CyPen enables slower, more uniform growth in nanocrystal syntheses, regulated by dynamic ligand binding. Moreover, the higher capacity of CyPen for dipolar interactions is expected to improve precursor solubilization, and stronger dispersive interactions with OAm and OAc ligands guarantee sufficient colloidal stability of the growing CsPbBr_3_ nanocrystals, overall contributing to a narrow nanocrystal size distribution and PL profile. Syntheses without antisolvent require considerably longer reaction times of many hours, limiting practical utility.

### 2.5 Photoluminescence Quantum Yield

**Figure 5:**
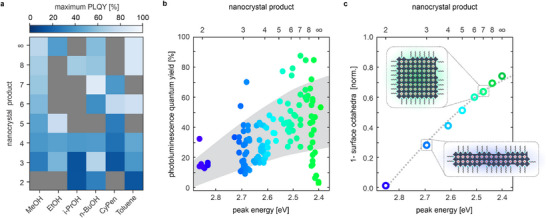
PLQY of CsPbBr_3_ nanocrystals. a) Maximum PLQY of pristine perovskite nanocrystals synthesized with MeOH, EtOH, i‐PrOH, n‐BuOH, toluene, and CyPen. The PLQY increases with a redshifted PL peak energy of the nanocrystal product for each antisolvent. Grey fields indicate no PLQY data for the respective product and antisolvent combination. b) Photoluminescence quantum yield (PLQY) of pristine perovskite nanocrystals with different PL peak energies, resulting from quantum confinement in up to three dimensions. c) Share of non‐surface PbBr_6_ octahedra in CsPbBr_3_ nanocrystals for a given PL peak energy and product, calculated based on nanocrystal dimensions determined by TEM and SAXS. The share of non‐surface PbBr_6_ octahedra follows the same trend as PLQY values in b). Insets show approximate dimensions of anisotropic 3ML NPLs and isotropic nanocubes.

With the knowledge that the selection of the antisolvent is crucial for obtaining desired nanocrystal products with narrow PL spectra, we examined whether this can be extended to the PLQY of pristine perovskite nanocrystals (**Figure** **5**a). Generally, the PLQY values increase with the size and emission wavelength of the perovskite nanocrystals. This is especially evident for CsPbBr_3_ nanocrystals synthesized with MeOH and toluene, whereby the former represents the most polar and the latter the least polar antisolvent tested. We confirm these observations by plotting the PLQY of all monodisperse samples in dependence on the PL peak energy (Figure 5b).

On average, the PLQY gradually increases from 15% for deep blue‐emitting 2ML NPLs to 60–70% for cube‐shaped nanocrystals with greenish emission. Despite the broader distribution of data points, this trend is also well reflected by the minimum and maximum PLQY values. PLQY predictions by the Synthesizer are in good agreement with experimental values, yet also rely on the accurate prediction of the PL peak wavelength (Figure S16, Supporting Information). We theorize that PLQY is independent of antisolvent and instead attribute the trend observed in Figure 5a,b to the size and shape‐dependent fraction of surface PbBr_6_ octahedra in different CsPbBr_3_ nanocrystal products obtained with this synthesis approach. As discussed before, TEM imaging and SAXS reveal a steady evolution from anisotropic to isotropic shape for CsPbBr_3_ nanocrystals with PL peak energies between 2.85 and 2.40 eV, i.e., from 2ML NPLs with large lateral sizes and smaller 3ML NPLs to approximately isotropic nanocubes (Figures S14 and S15, Supporting Information). The theoretical share of non‐surface PbBr_6_ octahedra is calculated based on experimentally derived dimensions for each nanocrystal product and depicted in Figure 5c, showing a strong correlation with the determined PLQY in Figure 5a. Together with the linewidth–lateral size relation, these trends constitute general design rules—maximize non‐surface octahedra fraction to raise PLQY and increase lateral extent at fixed thickness to minimize inhomogeneous broadening. In highly anisotropic CsPbBr_3_ NPLs, up to 100% of PbBr_6_ octahedra are located on the surface, while weakly confined nanocrystals possess merely 20–40% surface octahedra. CsPbBr_3_ nanocrystals with a higher fraction of surface octahedra likely exhibit more surface defects per volume. We purposely disregarded internal (bulk) defects, as their low densities will render most of the nanocrystals investigated here, with the possible exception of the largest nanocubes synthesized, void of them.^[^ ^67^, ^68^ ^]^ Here, halide vacancies are the most common type of defects; they promote non‐radiative over radiative recombination pathways and thereby result in a lower PLQY for defective nanocrystals.^[^ ^69^, ^70^ ^]^ The results from numerous syntheses in this project provide substantial statistical evidence, supporting the correlation between surface defects and PLQY as has been suggested for anisotropic perovskite nanocrystals.^[^ ^15^ ^]^ For weakly confined nanocrystals (nanocrystal product ∞), experimental PLQY values deviate more strongly from the expected values based on the share of non‐surface PbBr_6_ octahedra. This may be attributed to an increased likelihood of containing additional bulk defects that also enable non‐radiative recombination and lower PLQY values. Moreover, we note that the established two‐precursor synthesis approach employs PbBr_2_ as a single source of Pb and Br ions, inherently generating Br‐deficient growth conditions with a Pb/Br ratio of 1:2, compared to the approximate 1:3 stoichiometry in the emissive, 3D CsPbBr_3_ perovskite phase, or even Br‐rich conditions in other three‐precursor synthesis approaches.^[^ ^60^, ^62^, ^71^ ^]^ Nevertheless, slightly Br‐deficient syntheses still enable the formation of stable CsPbBr_3_ perovskite, and resulting surface halide vacancies can be effectively passivated with facile post‐synthesis treatments. To verify this, we employed the commonly used enhancement of pristine CsPbBr_3_ nanocrystal samples with a solution of PbBr_2_, OAm, and OAc.^[^ ^39^, ^72^ ^]^ All samples showed remarkably improved PLQY values (Figure S17, Supporting Information) and larger nanocrystal products even approach near‐unity values while maintaining the characteristic peak wavelength and narrow emission profile. Overall, the results here show an unprecedented, simultaneous control of emission wavelength and narrow PL profiles, while exhbiting good to excellent quantum yields.^[^ ^73^, ^74^, ^75^ ^]^

### 2.6 Extension to CsPbI_3_ Nanocrystals

Lastly, we highlight the potential of the Synthesizer for synthesis optimization in other materials by demonstrating the transferability of our approach to a related perovskite system. For this, we synthesized and characterized CsPbI_3_ nanocrystals in ambient conditions by following a similar procedure of ligand‐assisted spontaneous crystallization in non‐polar solvents.^[^ ^76^ ^]^ In this material system, we also varied the molar Cs/PbI_2_ precursor ratio in the range between 0.0‐ 1.0 but omitted the subsequent addition of antisolvent to avoid phase transformation to the non‐emissive δ‐CsPbI_3_ phase.^[^ ^77^ ^]^

**Figure 6:**
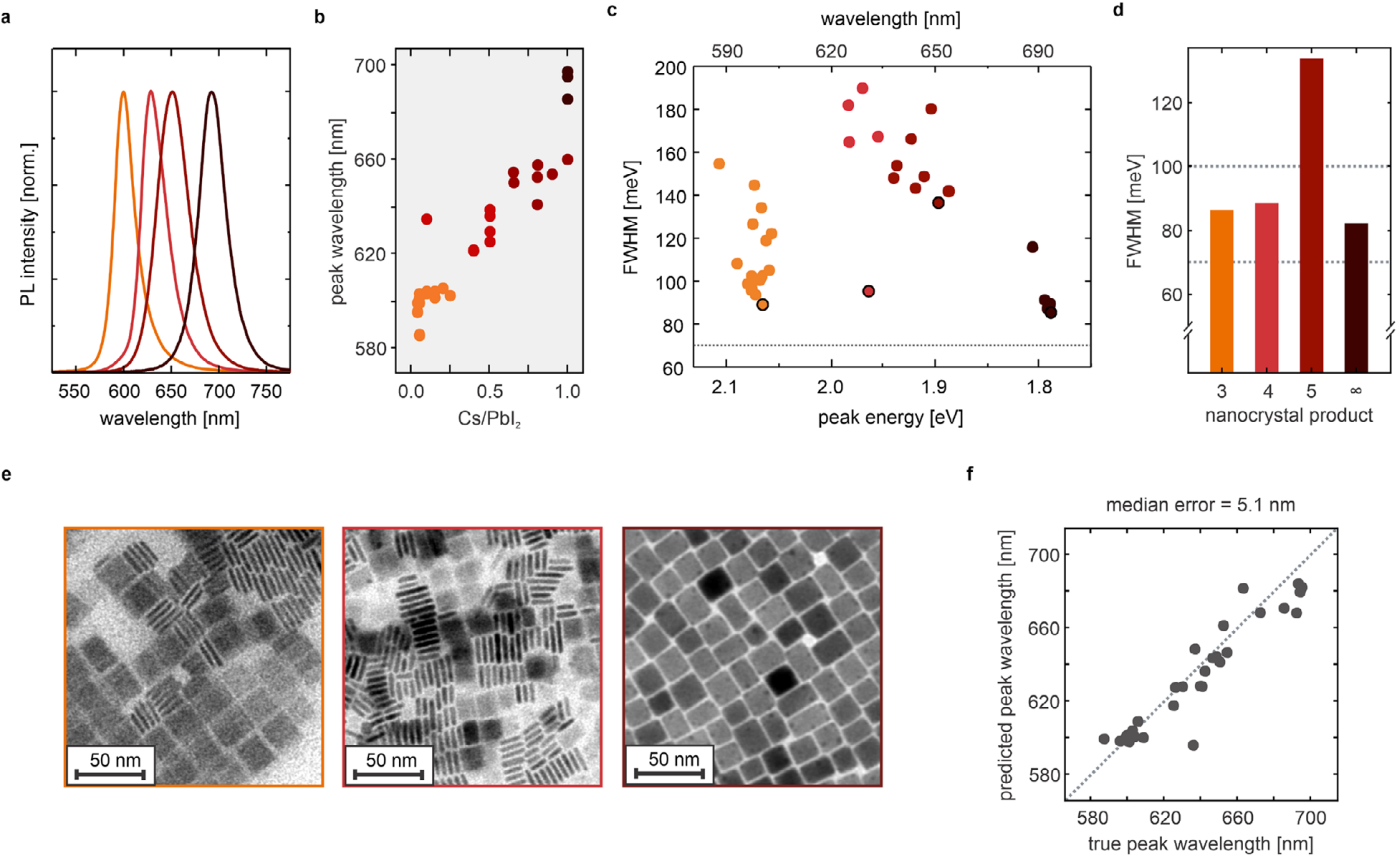
Application of the Synthesizer to the synthesis CsPbI_3_ nanocrystals. a) PL spectra of isolated CsPbI_3_ nanocrystal ensembles, synthesized by ligand‐assisted spontaneous crystallization in nonpolar solvents and ambient conditions. The depicted spectra with PL peak wavelengths at 598, 630, 650, and 695 nm are representative of nanocrystal products 3, 4, 5, and ∞. b) Variation of the molar Cs/PbI_2_ precursor ratio enables the synthesis of CsPbI_3_ nanocrystals with tunable emission, covering the orange to deep‐red spectral range between 583 and 695 nm. c) Distribution of FWHM values concerning the PL peak energy (wavelength). The narrowest PL spectra were observed at the lowest and highest PL peak energies, whereas nanocrystals with intermediate PL peak energies exhibit broader spectra on average. d) Optimized CsPbI_3_ nanocrystals exhibit narrow PL profiles, with FWHM values between 82 and 139 meV. e) Exemplary TEM images of CsPbI_3_ nanocrystal products 3, 4, and ∞, to which we assign to 3ML, 4ML NPL, and cube‐shaped morphologies. Edge lengths amount to (20.9 ± 2.4) nm, (24.9 ± 2.7) nm, and (18.4 ± 2.7) nm, respectively. f) LOO regression plot for the PL peak wavelength prediction of CsPbI_3_ nanocrystals, based on the molar Cs/PbI_2_ precursor ratio in the synthesis. Even for a limited data set, a comparison between true and predicted PL peak wavelength yields a low median error of ± 5 nm and a mean error of ± 8 nm, confirming a good prediction accuracy by the Synthesizer.

These preliminary syntheses produced different CsPbI_3_ nanocrystal products with tunable orange to deep‐red emission between 583‐695 nm, as shown by the representative PL spectra in **Figure** **6**a,b. Similar to CsPbBr_3_, the PL peak wavelength of CsPbI_3_ redshifts with increasing Cs/PbI_2_ precursor ratio, allowing for reasonable emission control beyond what has been established for other iodide‐based perovskite materials.^[^ ^78^ ^]^ There is a noticeable clustering of the PL peak wavelength (or energy) of the synthesized CsPbI_3_ nanocrystals around characteristic values, which we assign to nanocrystal products 3, 4, 5, and ∞, as defined in Table S6 (Supporting Information). FWHM values of optimized CsPbI_3_ nanocrystals in Figure 6c,d are narrow, ranging from 82 to 139 meV, with a thickness dependence exhibiting high similarity to the CsPbBr_3_ results. Accordingly, the narrowest linewidths are obtained for the lowest and highest PL peak energy, i.e., nanocrystal products 3 and ∞. These observations suggest that the underlying nanocrystal morphology is similar, implying a shared nanocrystal formation mechanism. Indeed, TEM analysis of representative samples for nanocrystal products 3, 4, and ∞—shown in Figure 6e—reveals an anisotropic 3ML, 4ML NPL morphology for orange‐ and red‐emitting samples, whereas deep red‐emitting nanocrystals have a cuboid shape. Further optimization is likely achievable via lateral size control, as demonstrated for CsPbBr_3_ NPLs; however, these CsPbI_3_ NPLs already satisfy the Rec.2020 standard.^[^ ^79^ ^]^ Synthesis parameters for optimized CsPbI_3_ nanocrystals are given in Table S7, Supporting Information.

Even based on a limited data set of 35 syntheses, the Synthesizer regression allows for a precise prediction of the PL peak wavelength with overall ±5 nm (median error) or ±8 nm (mean error) accuracy, as confirmed by the LOO regression plot in Figure 6f. This forms an ideal and reliable foundation for the targeted optimization of CsPbI_3_ nanocrystals concerning peak narrowness and PLQY, employing the Synthesizer to identify ideal synthetic parameters. Beyond halide perovskites, the principle‐based design of Synthesizer is readily adaptable to chemically distinct systems, for example, I–III–VI semiconductor quantum dots, where it could accelerate identification of key synthetic parameters and guide optimization.

## 3 Conclusion

In this work, we introduced the Synthesizer, a machine learning‐guided framework for optimizing the optical properties of colloidal CsPbBr_3_ nanocrystals synthesized under ambient conditions. By systematically varying precursor ratios and incorporating a geometric encoding scheme for antisolvent molecules, we achieve nanometer‐level precision in tuning PL peak wavelength across the 430–520 nm range—a critical range for color‐specific optoelectronic applications. Our methodology further enables predictive control over spectral linewidths (FWHM reduced to 70–100 meV) and PLQY, revealing design principles that link synthesis parameters to surface defect densities. Together, these three properties—color, color purity, and emission efficiency—define the functional performance of perovskite emitters. The values achieved here are among the most finely tuned and reproducible reported to date for blue‐to‐green perovskite nanocrystals synthesized under ambient conditions. This high accuracy, joint optimization represents a significant advance toward rational synthesis design. In addition, by identifying the as/PbBr_2_ ratio as a novel and critical tuning parameter alongside the established Cs/PbBr_2_ ratio, our study provides mechanistic handles that enable a more quantitative discussion of antisolvent‐accelerated nanocrystal growth.

Synthesizer also demonstrated robust reproducibility across different experimenters and local conditions (temperature, humidity), yielding nearly identical optical properties. This underscores its practical usability and paves the way for broader implementation by other groups, enabling both reproduction and innovation in nanocrystal design. Unlike autonomous robotic or microfluidic systems, Synthesizer requires no specialized hardware, making ML‐guided optimization immediately deployable in typical academic and industrial labs. We therefore view it as a complementary path that broadens access to data‐driven materials discovery. Beyond halide perovskites, the Synthesizer offers a generalizable, data‐efficient platform for guided synthesis exploration. We demonstrate its adaptability by accurately predicting outcomes for untested antisolvents and for a new material system (CsPbI_3_). Its flexibility in incorporating additional synthesis variables, such as temperature, ligand composition, and reaction dynamics, positions it as a foundational tool for automated, high‐throughput synthesis pipelines. Although the machine learning methods employed are well established, their integration with chemically meaningful encodings and physical synthesis constraints results in a framework that is both practically usable and adaptable to new material systems. While we demonstrate this with CsPbI_3_, the principle‐based design of Synthesizer allows straightforward extension to other nanocrystal families, for example I–III–VI quantum dots, thereby underscoring its broader potential. With publicly available code and datasets, this framework can serve as a starting point for collaborative development and deployment across material classes. Ultimately, we envision the Synthesizer accelerating the targeted optimization of next‐generation nanomaterials for renewable energy, photonics, and beyond.

## 4 Experimental Section

### Nanocrystal Synthesis and Characterization

### Cs‐Oleate Precursor

A Cs‐oleate precursor (0.02 m) was prepared by dissolving Cs_2_CO_3_ (0.1 mmol, 32.6 mg) in OAc (10 mL) while stirring at 85 °C for up to 3 h until a clear solution was obtained. A higher concentration Cs‐oleate precursor (0.2 m) was prepared by dissolving Cs_2_CO_3_ (1.0 mmol, 326 mg) in OAc (10 mL) while stirring at 85 °C for up to 3 h until a clear solution was obtained. Depending on the synthesis parameters, the Cs‐oleate precursor was used as prepared or diluted with additional oleic acid to adjust the Cs‐oleate concentration as required.

### PbX_2_ Precursor

Similarly, the PbBr_2_ precursor (0.01 m) was prepared by dissolving PbBr_2_ (0.1 mmol, 36.7 mg) in toluene (9.8 mL), oleic acid, and oleylamine (100 μL each) under stirring at 85 °C for up to 3 h until a clear solution was obtained. A higher concentration PbBr_2_ precursor (0.1 m) was prepared by dissolving PbBr_2_ (1.0 mmol, 367 mg) in toluene (8 mL), OAc, and OAm (1 mL each) while stirring at 85 °C for up to three hours until a clear solution was obtained. A PbI_2_ precursor (0.01 m) was prepared by dissolving PbI_2_ (0.1 mmol, 46.1 mg) in toluene (9.8 mL), OAc, and OAm (100 μL each) under stirring at 85 °C for up to three hours until a clear solution was obtained. Depending on the synthesis parameters, the PbX_2_ precursor was used as prepared or diluted with additional toluene to adjust the PbX_2_ concentration as required. Precursors were stored in ambient conditions.

### Synthesis of CsPbBr_3_ Nanocrystals

Syntheses were carried out in ambient conditions (air, 20‐40% humidity, *T* = 20–25 °C). A glass vial was charged with PbBr_2_ precursor, and Cs‐oleate was injected while stirring vigorously. Then, the antisolvent was added after 10–15 s. The reaction mixture was stirred until precipitation of perovskite nanocrystals occurred and was then centrifuged (3 min, 2097 × *g*). The precipitate (P), which contains CsPbBr_3_ nanocrystals, was redispersed in 1 mL n‐hexane and kept for further characterization. The supernatant (S) was discarded if non‐emissive and kept for further characterization if emissive. In this case, the perovskite nanocrystals in the supernatant can be precipitated with the addition of 2:1 methyl acetate and isolated by centrifugation (3 min, 2097 × *g*), followed by redispersion in 1 mL of n‐hexane. Insoluble byproducts or bulk perovskite material in redispersed samples were removed in an additional centrifugation step (3 min, 2097 × *g*), if necessary.

### Synthesis of CsPbI_3_ Nanocrystals

A glass vial was charged with PbI_2_ precursor, and Cs‐oleate was injected while stirring vigorously. The reaction mixture was stirred until perovskite nanocrystal precipitation occurred and then centrifuged (3 min, 15856 × *g*). The supernatant (S) was discarded if non‐emissive and kept for further characterization if emissive. The red precipitate (P), which contains CsPbI_3_ nanocrystals, was redispersed in 1 mL n‐hexane and kept for further characterization. Unsoluble byproducts in redispersed samples were removed in a second centrifugation step (3 min, 2097 × g), if necessary.

### Photoluminescence Spectroscopy

Optical characterization was performed immediately after synthesis and purification. PL spectra of pristine colloidal CsPbX_3_ nanocrystals were measured with a commercial FluoroMax‐4Plus spectrometer equipped with a xenon arc lamp and an F‐3031 transmission accessory (HORIBA Scientific). For CsPbBr_3_ nanocrystals, the excitation wavelength was set to 380 nm, and spectra were recorded in the range between 400 and 600 nm. Absolute PLQY was determined in a Quanta‐Phi F‐3029 integrating sphere (HORIBA Scientific) with direct excitation. The optical density of the colloidal samples was adjusted to ⩽ 0.1 at the excitation wavelength of 380 nm. For CsPbI_3_ nanocrystals, the excitation wavelength was set to 480 nm, and spectra were recorded in the range between 500 and 800 nm.

### Transmission Electron Microscopy

TEM images were recorded on a JEOL JEM‐1100 microscope operated at an acceleration voltage of 80 kV. Specimen preparation was carried out by dropcasting 5–10 μL of colloidal perovskite solutions on TEM grids (Electron Microscopy Sciences, Cu with 10/1 nm Formvar/carbon). Nanocrystal structures were drawn using VESTA.^[^ ^80^ ^]^

### Data Classification and Analysis

Subsequent to optical characterization, the PL data were classified based on three criteria: emissivity, purification, and monodispersity. First, PL profiles were analyzed to confirm the presence of a peak with a signal‐to‐noise ratio (SNR) ⩾ 5:1, comparing maximum PL intensity in the emission region to baseline noise from a non‐emissive region. Samples below this threshold were labeled non‐emissive (0), otherwise as emissive (1). Second, each sample was labeled based on whether CsPbBr_3_ nanocrystals were isolated from the supernatant (S) or precipitate (P) following the purification step by centrifugation. Third, PL profiles were assessed using the built‐in *PeakAnalyzer* tool of the spectrometer software (FluorEssence V3.8.01, constant baseline, local maximum method). Samples were labelled as monodisperse (1) if they exhibited a single, well‐defined peak with FWHM ⩽30 nm, based on practical spectral purity standards for narrowband emission.^[^ ^45^, ^46^ ^]^ If multiple peaks or excessive peak broadening (FWHM >30 nm) were observed, the sample was classified as polydisperse (0). All assigned labels were added to the central dataset and served as filters in the Synthesizer code to enable adequate data selection.

For data analysis, the PL peak wavelength was extracted as the global maximum of the respective PL spectrum, given in wavelength units. To obtain accurate and comparable FWHM values across the entire wavelength range, PL spectra were converted from wavelength to eV via Jacobian conversion, and the FWHM was determined by performing linear interpolation of the experimental data points.^[^ ^81^ ^]^

### Machine Learning

The machine learning models used in this study (GP regression, Bayesian optimization) are well established; however, our contribution lies in adapting them to a real synthesis space–chemically meaningful, noisy, low‐data, and constrained. Domain knowledge, such as antisolvent geometry encodings and synthesis heuristics, was incorporated and validated against physical experimental outcomes.

### Data Selection

Different data selection requirements were applied to the starting data set, depending on the specific synthesis request. For prediction and optimization of PL peak wavelength, peak narrowness or FWHM, and PLQY, non‐emissive samples and polydisperse samples with multiple PL peaks were standardly excluded from the data set. If emissive CsPbBr_3_ perovskite nanocrystals were found both in the precipitate (P‐type) and the supernatant (S‐type) of a synthesis, a comparison revealed minor differences in the peak PL wavelength of the CsPbBr_3_ nanocrystals in each fraction. Therefore, P‐type and S‐type data were treated separately for PL peak wavelength predictions (Figure 2). For FWHM minimization (Figure 3) and PLQY analysis (Figure 5), both S‐ and P‐type data were included.

### Feature Selection

The initial data set contained a large number of synthesis parameters, of which only a subset was actually correlated with any given regression target. In general, low‐level features such as volumes and concentrations were not very expressive parameters for nanocrystal synthesis. Instead, calculated quantities, such as molar amounts of substance and precursor ratios, defined the chemical reaction processes more accurately. The approach chosen in this work consisted of alternately removing uncorrelated synthesis parameters and calculating high‐level features from the remaining parameters to arrive at a small set of expressive and strongly correlated parameters.

### Gaussian Processes

Based on the high‐level synthesis parameters, all target predictions were made using Gaussian Processes implemented through the GPRegression model from the GPy library.^[^ ^82^ ^]^ This nonparametric, probabilistic type of model did not require any predefined functional form and therefore no initial information about the underlying dependencies or physical mechanisms. Contrary to more complex models, such as neural networks, Gaussian Processes generally exhibited much better performance on small datasets as well as uncertainty awareness, and naturally account for noise in the data. In this case, there was no reason for the system to exhibit variable noise, and therefore, this was accounted for by using a standard noise kernel with a fixed variance and trainable mean. Additionally, the fully trained and optimized models, as well as their kernel and hyperparameters, were highly interpretable and allow visualization of the learned mapping, which can give valuable insights into the underlying chemical and physical processes.

### Gaussian Process Parameters

The parameters required for the GP model were a kernel, or covariance function, and a mean functional, which fully defined the covariance and consequently the prior in this framework. The kernel acted as a similarity measure between points in parameter space and thus encoded how correlation decays over distance. The mean was defining a logical value around which the predictions loosely fluctuate. In this work, the data exhibited a variety of sharp changes and small, localized features like plateaus and steps. As the estimated function in a Gaussian Process typically reflected the smoothness properties of the chosen kernel, the commonly used RBF (radial basis function) kernel failed to capture these sharp features due to its infinite differentiability. In contrast, the non‐differentiable exponential kernel with a sharp decay over distance was particularly well‐suited for modeling the step‐like behavior observed here. Its standard implementation in the GPy library was used as:

(1) $$k\left(x,x^{\prime },\sigma ,l\right)=\sigma^{2}\text{exp}\left(-\frac{|x-x^{\prime }|}{l}\right)$$

with amplitude σ, length scale *l* and metric |*x* − *x*′|. The GPy library offered a choice between an isotropic and anisotropic implementation, where the length scale *l* was either the same for all dimensions or was optimized independently for each dimension. Here, the isotropic version has shown better performance, possibly because of the reduced number of trainable parameters. The kernel parameters were determined by optimizing the marginal logarithmic likelihood. The mean was chosen as the zero mean, as the one providing the least bias.

### Iterative Optimization

To find the ideal synthesis parameters, points in a predefined search space, set by the box constraints in Table S1 (Supporting Information) were sampled and evaluated by a set of predictors and assigned a quality score through an objective function. Additionally, the molar ratios Cs/PbBr_2_ and as/PbBr_2_ were constrained to a reasonable search space of (0.0,1.0) and (0.0,10^4^), respectively. The quality score then informed the next sampling step in a Bayesian framework implemented through the GPyOpt library.^[^ ^83^ ^]^ Initially, the focus was the acquisition of points that inject the most amount of information into our model. This resulted in data‐efficient training and reduced the experimental effort before achieving a well behaving model. This can be realized by sampling points whose prediction exhibited the highest uncertainty and therefore were the most agnostic for the model. Once the overall uncertainty had decreased, the authors were free to sample in areas of promising material property prediction, trusting that the sampled points capture the entire physical space. It must be noted that the objective switch between exploration of points and exploitation of knowledge is gradual. In practice, GP uncertainty estimated guided this balance: early sampling prioritized high‐uncertainty regions to improve global model accuracy, while later iterations shifted toward expected improvement in promising regions. This ensured that the resulting predictor was both globally reliable and sample‐efficient, rather than narrowly tuned to a single optimum. In practice, acquisition prioritized max‐uncertainty candidates early to de‐risk extrapolation and reduce overfitting, before shifting to expected‐improvement in regions meeting multi‐objective constraints (PL peak wavelength, FWHM, PLQY). LOO calibration and transfer errors were reported alongside predictions to quantify decision risk during acquisition.

### Baseline Data

Data efficiency was crucial when designing a predictor in this framework, where the main challenge lies in constraining the Gaussian Process at the parameter space boundaries, even with sparse data. Specific antisolvent‐independent data points at the boundaries were identified on the basis of chemical models and experimental data and were added to every individual data set. Specifically, the experimental no‐antisolvent data and extrapolated bulk perovskite limit at Cs/PbBr_2_ = 1.0 ratio formed the ideal limits for every antisolvent and strongly reduced the necessary data for accurate predictions. They were strictly excluded from the test data for LOO accuracy scores.

## Conflict of Interest

The authors declare no conflict of interest.

## Supporting information

Supporting Information

## Data Availability

The code, documentation, and instructions for adaptation to other use cases, as well as the data set for this project, are available from github.com/leoluber/synthesizer.
