# Supplementary material for: Synthesizer: Chemistry‐Aware Machine Learning for Precision Control of Nanocrystal Growth
Source: Adv Mater. 2025 Nov 5;38(4):e09472. doi: 10.1002/adma.202509472 (PMC12810630; doi:10.1002/adma.202509472)
Supplement: Supplementary file 1 — Supporting Information [file ADMA-38-e09472-s001.pdf]

ADVANCED MATERIALS

Supporting Information

for *Adv. Mater.*, DOI 10.1002/adma.202509472

Synthesizer: Chemistry-Aware Machine Learning for Precision Control of Nanocrystal Growth

*Nina A. Henke, Leo Luber, Ioannis Kouroudis, Jonathan Paul, Alexander Schuhbeck, Lukas M. Rescher, Tizian Lorenzen, Veronika Mayer, Knut Müller-Caspary, Bert Nickel, Alessio Gagliardi and Alexander S. Urban**

Supporting Information

Synthesizer: Chemistry-Aware Machine Learning for Precision Control of Nanocrystal Growth

Nina A. Henke[1,†], *Leo Luber*[1,†], *Ioannis Kouroudis*[2,†], *Jonathan Paul*[1], *Alexander Schuhbeck*[1], *Lukas M. Rescher*[3], *Tizian Lorenzen*[4], *Veronika Mayer*[1], *Knut Müller-Caspary*[4], *Bert Nickel*[3], *Alessio Gagliardi*[2], *Alexander S. Urban*[1,*]

[1] N. A. Henke, L. Luber, J. Paul, A. Schuhbeck, V. Mayer, Prof. A. S. Urban
Nanospectroscopy Group and Center for NanoScience (CeNS), Nanoinstitute Munich
Department of Physics
Ludwig-Maximilians-Universität (LMU) München, 80539 Munich, Germany

[2] I. Kouroudis, Prof. A. Gagliardi
Chair of Simulation of Nanosystems for Energy Conversion, Department of Electrical Engineering
TUM School of Computation, Information, Technology, Atomistic Modeling Center (AMC), Munich Data Science Institute (MDSI)
Technical University Munich (TUM), 85748 Garching, Germany

[3] L. M. Rescher, Dr. B. Nickel
Soft Condensed Matter Group and Center for NanoScience (CeNS), Department of Physics
Ludwig-Maximilians-Universität (LMU) München, 80539 Munich, Germany

[4] T. Lorenzen, Prof. K. Müller-Caspary
Department of Chemistry and Center for NanoScience (CeNS)
Ludwig-Maximilians-Universität (LMU) München, 81377 Munich, Germany

E-mail: urban@lmu.de

† These authors contributed equally.

1 Materials

Cesium carbonate (Cs_2CO_3, 99%, Sigma Aldrich), lead bromide ($PbBr_2$, 98%, Sigma Aldrich), lead iodide (PbI_2, 99%, Alfa Aesar), oleic acid (OAc, technical grade, 90%, Sigma Aldrich), oleylamine (OAm, technical grade, 70%, Sigma Aldrich), toluene (99.9%, VWR Chemicals), n-hexane (97%, VWR Chemicals), methanol (MeOH, 99.8%, Sigma Aldrich), ethanol (EtOH, 99.5%, Sigma Aldrich), isopropanol (i-PrOH, for analysis, Merck), n-butanol (n-BuOH, 99%, Sigma Aldrich), cyclopentanone (CyPen, for synthesis, Merck), methyl acetate (99%, for synthesis, Merck). All chemicals listed above were used as received without further purification.

2 Syntheses

Supporting Table 1: Constraints for the synthesis of $CsPbBr_3$ nanocrystals. Summary of experimental constraints for the preparation of perovskite nanocrystals by antisolvent-assisted spontaneous crystallization.

experimental constraint	notes
$OAm/PbBr_2 = 2.1$	fixed ligand ratio
$OAc/PbBr_2 = 2.8$	fixed ligand ratio
$0.020\ M \leq c_{Cs} \leq 0.200\ M$	precursor concentration
$0.001\ M \leq c_{PbBr_2} \leq 0.100\ M$	precursor concentration
$50\ µL \leq V_{Cs} \leq 5000\ µL$	pipetting
$500\ µL \leq V_{PbBr_2} \leq 5000\ µL$	pipetting
$0 \leq V_{as} \leq 5000\ µL$	pipetting
$V_{Cs} + V_{PbBr_2} + V_{as} \leq 10\ mL$	reaction vial

Supporting Table 2: Classification of $CsPbBr_3$ nanocrystals. Perovskite nanocrystal were sorted into different groups according to the PL peak wavelength or PL peak energy. The upper and lower limit for each nanocrystal product is listed.

	lower limit [nm]	upper limit [nm]	upper limit [eV]	lower limit [eV]
2	429	437	2.89	2.84
3	455	465	2.72	2.67
4	470	480	2.63	2.58
5	481	489	2.58	2.54
6	490	497	2.53	2.49
7	498	504	2.49	2.46
8	505	509	2.46	2.44
∞	510	520	2.43	2.38

Supporting Table 3: Summary of syntheses. Nanocrystal syntheses with different antisolvents are listed, including details on number of syntheses with monodisperse, polydisperse and non-emissive samples as products. The label 'polydisperse' is used to describe synthesis products which exhibit multiple, or severely broadened emission peaks.

antisolvent	syntheses	monodisperse	polydisperse	non-emissive
MeOH	65	58	6	1
EtOH	71	60	8	3
i-PrOH	33	26	7	0
n-BuOH	45	35	10	0
CyPen	57	56	1	0
toluene	16	15	1	0

Supporting Table 4: Optimized synthesis parameters for each $CsPbBr_3$ nanocrystal product. Precursor concentrations, volumes and as well as antisolvent type and volume for the synthesis of optimized nanocrystal products with the narrowest PL profiles. Syntheses are marked with symbols corresponding to the optimization method: $\star$ for direct *Synthesizer* suggestion, c for confinement tuning and a for optimization by antisolvent choice.

nanocrystal product	V_{PbBr_2} [µL]	c_{PbBr_2} [M]	V_{Cs} [µL]	c_{Cs} [M]	antisolvent	V_{as}[(µL]	FWHM [meV]
2	2385	0.074	68	0.020	n-BuOH	2235	69 ($\star$)
3	1000	0.010	100	0.045	-	0	73 (c)
4	2250	0.007	68	0.020	CyPen	2520	87 ($\star, c, a$)
5	1500	0.020	150	0.020	EtOH	2000	98
6	2590	0.010	403	0.020	CyPen	5000	96 ($\star, a$)
7	750	0.007	75	0.020	CyPen	2000	87 (a)
8	1000	0.003	100	0.020	CyPen	1335	80 (a)
∞	695	0.010	249	0.020	MeOH	1155	75

3 Prediction of Photoluminescence Peak Wavelength

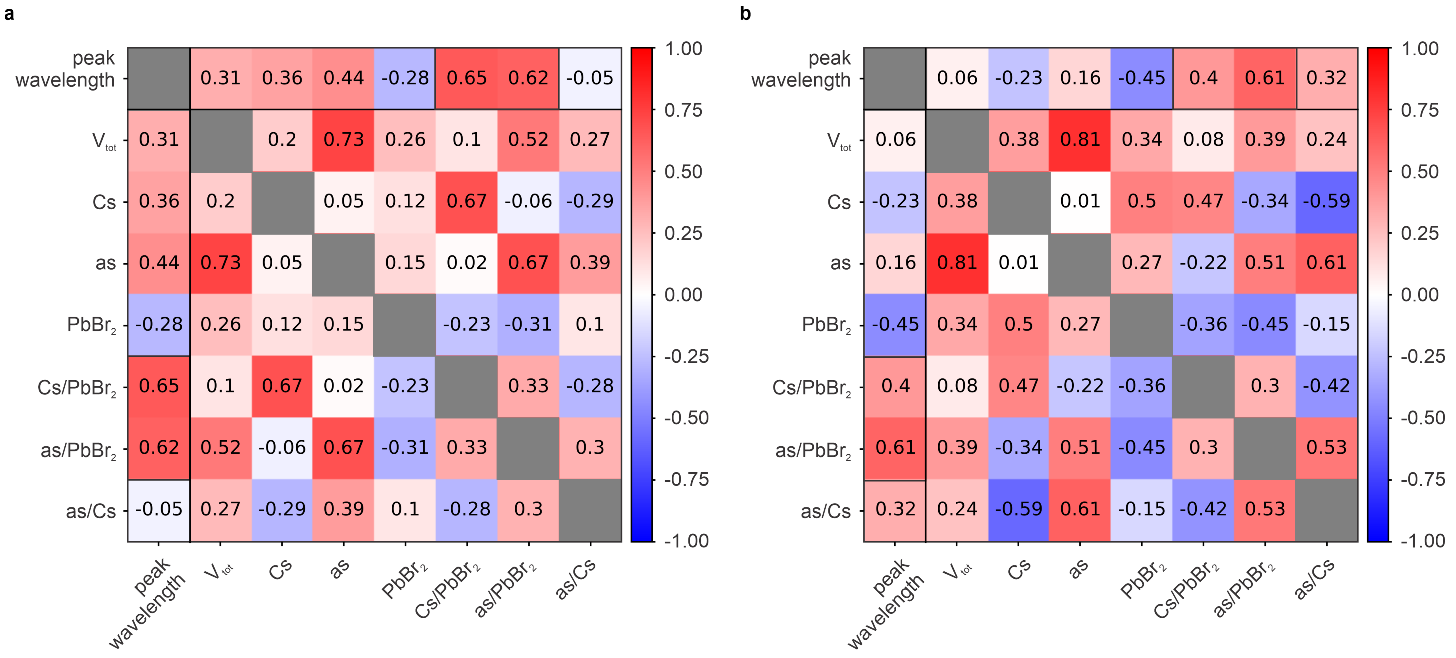

Supporting Figure 1: Correlation matrix for feature selection. A selection of high level calculated features is compared in regard to their correlation with the PL peak position of the $CsPbBr_3$ nanocrystal product in the a) precipitate (P) or b) supernatant (S). In general, we find that the relative molar ratios are more expressive than the individual, absolute molar values or the total volume. However, the as/Cs ratio and PL peak position show no noteworthy correlation and can be omitted. Therefore, the remaining two ratios $Cs/PbBr_2$ and $as/PbBr_2$ were identified as the best parameters for PL peak prediction, resulting in a reduced, two-dimensional synthesis parameter space. This systematic approach of feature selection on calculated properties gives some initial insights into the underlying chemical processes. Components whose ratios between amounts of substance correlate with the type of product are likely to interact during the synthesis.

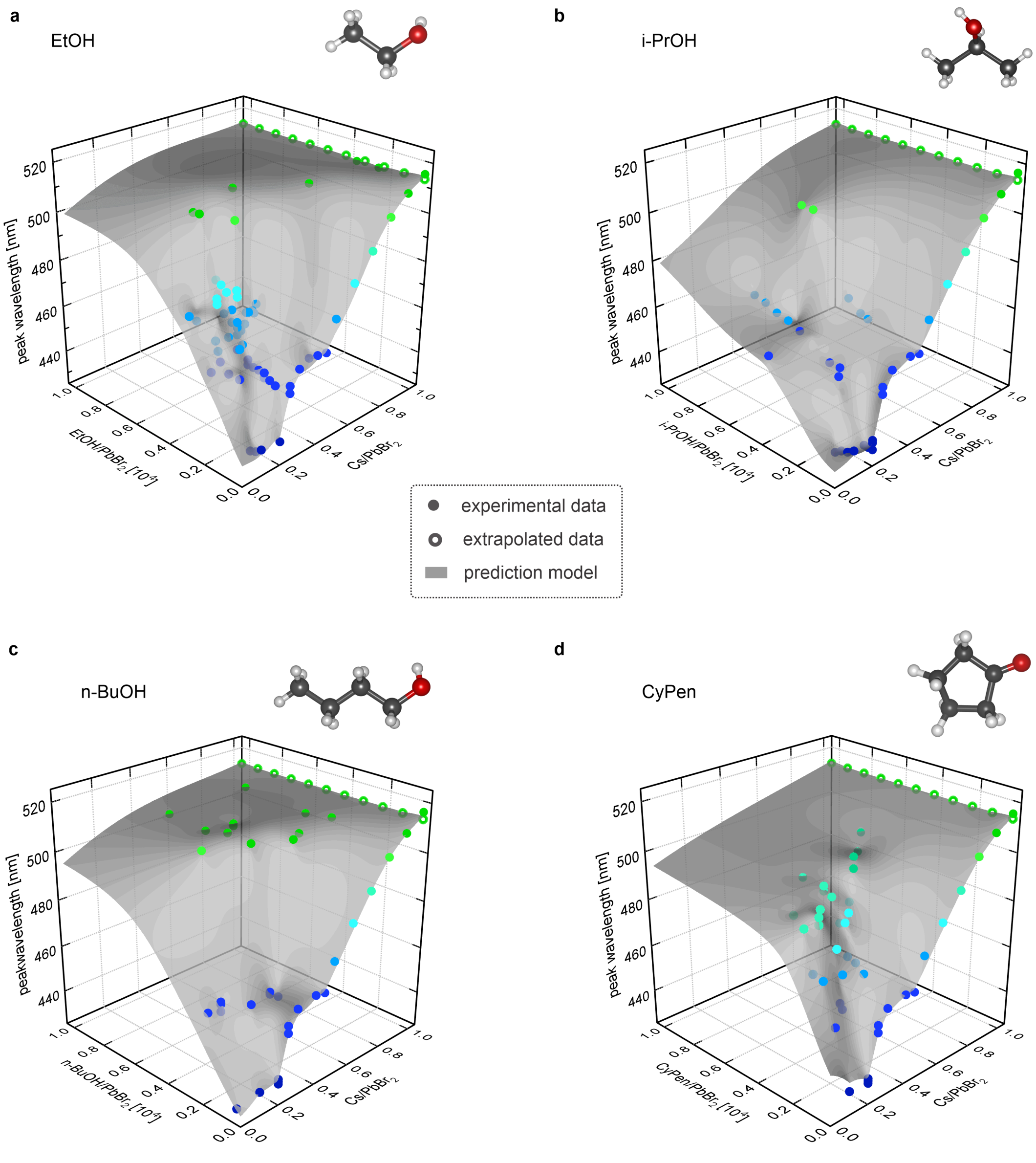

Supporting Figure 2: Three-dimensional representation of parameter space and PL peak wavelength for P-type samples synthesized with EtOH, i-PrOH, n-BuOH and CyPen antisolvent. Two-dimensional parameter space defined by Cs/$PbBr_2$ and antisolvent/$PbBr_2$ ratio with corresponding true PL peak wavelengths (colored dots) and the fully trained regression model (dark grey surface), including antisolvent-independent data at antisolvent/$PbBr_2$ = 0.0 and extrapolated data at Cs/$PbBr_2$ = 1.0 from the baseline data frame. Overall, increasing the antisolvent/$PbBr_2$ ratio results in a further redshifted PL peak wavelength for a) EtOH, b) i-PrOH, c) n-BuOH and d) CyPen. The transition from blue- to green-emitting nanocrystal products is steeper for alcohol antisolvents, especially n-BuOH, and more gradual for the ketone antisolvent, CyPen. Syntheses with i-PrOH also require a minimum Cs/$PbBr_2$ ratio of ≥ 0.4 to yield green-emitting $CsPbBr_3$ nanocrystals.

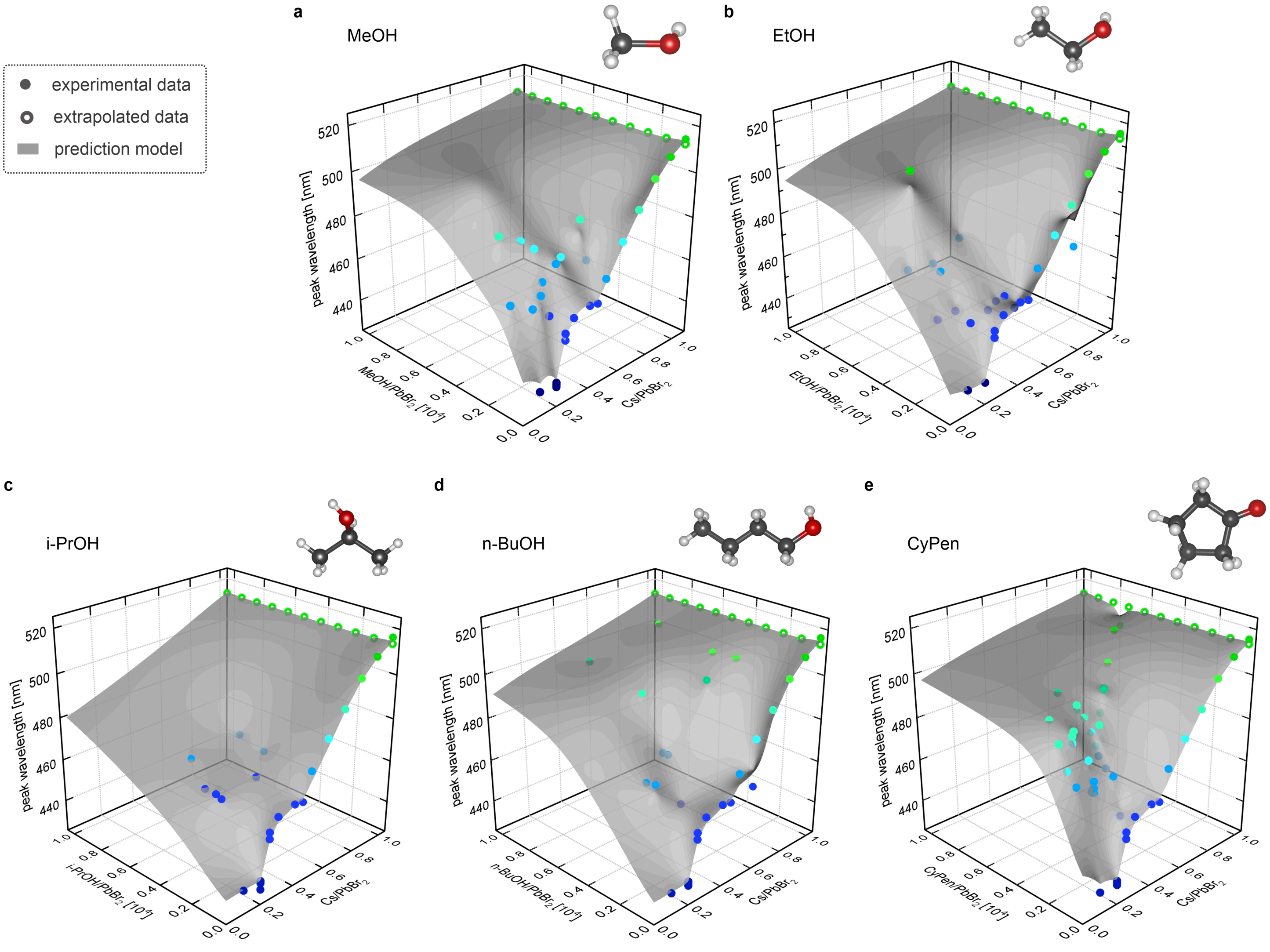

Supporting Figure 3: Three-dimensional representation of parameter space and PL peak wavelength for S-type samples. Two-dimensional parameter space defined by $Cs/PbBr_2$ and antisolvent/$PbBr_2$ ratio with corresponding true PL peak wavelengths (colored dots) and the fully trained regression model (dark grey surface), including antisolvent-independent data at antisolvent/$PbBr_2$ = 0.0 and extrapolated data at $Cs/PbBr_2$ = 1.0 from the baseline data frame. Similarly to P-type data, increasing the antisolvent/$PbBr_2$ ratio results in a further redshifted PL peak wavelength for a) MeOH, b) EtOH, c) i-PrOH, d) n-BuOH and e) CyPen. The transition from blue- to green-emitting nanocrystal products is steeper for alcohol antisolvents, especially n-BuOH, and more gradual for the ketone antisolvent, CyPen.

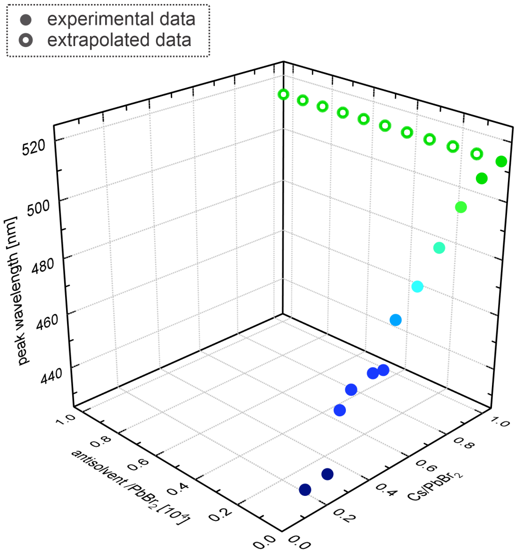

Supporting Figure 4: Antisolvent independent baseline data. This set of experimental and extrapolated datapoints at the boundaries of the parameter space is inherently antisolvent independent and can therefore be added to the data set for any antisolvent. As the PL peak wavelength is assumed to be continuous, the experimental data points from syntheses with $Cs/PbBr_2$ = 0.0-1.0 but without antisolvent represent the lower limit for any antisolvent molecule. The upper limit at $Cs/PbBr_2$ = 1.0 is given by the bulk emission wavelength of $CsPbBr_3$. This approach has proven critical to confine the Gaussian Process model in low data regimes and has significantly improved the prediction accuracy. These data points have been strictly excluded from test data for LOO (Leave-One-Out) accuracy scores.

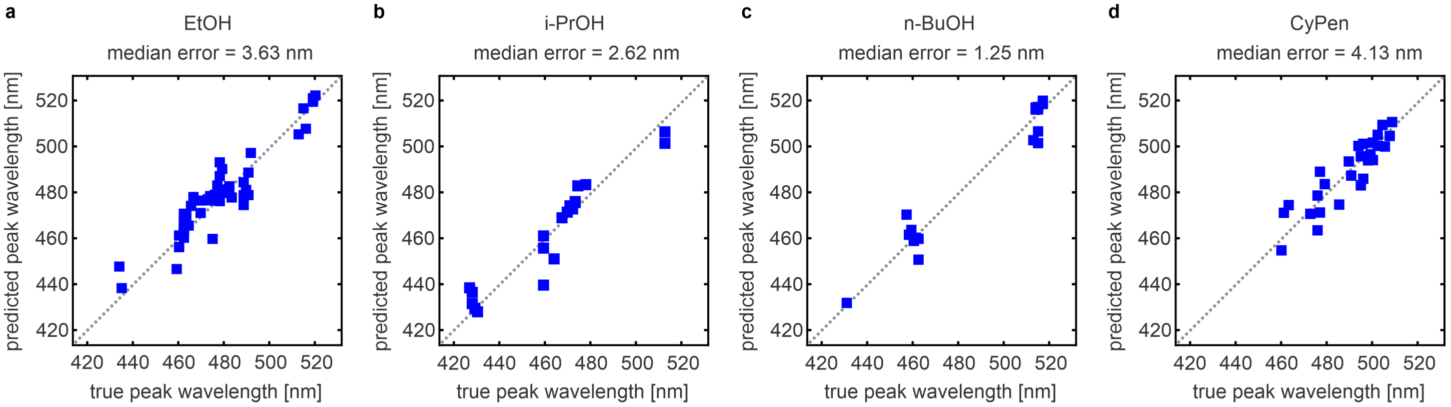

Supporting Figure 5: Prediction accuracy of photoluminescence (PL) peak wavelength for P-type data. LOO regression plots for PL peak wavelength prediction in syntheses with different antisolvents, shown for a) EtOH, b) i-PrOH, c) n-BuOH and d) CyPen. A comparison between true PL peak wavelength and predicted PL peak wavelength yields low median errors ($< \pm$ 4 nm) and confirms a high prediction accuracy for all antisolvents. The mean errors are 4.7 nm (EtOH), 5.0 nm (i-PrOH), 3.4 nm (n-BuOH) and 5.2 nm (CyPen).

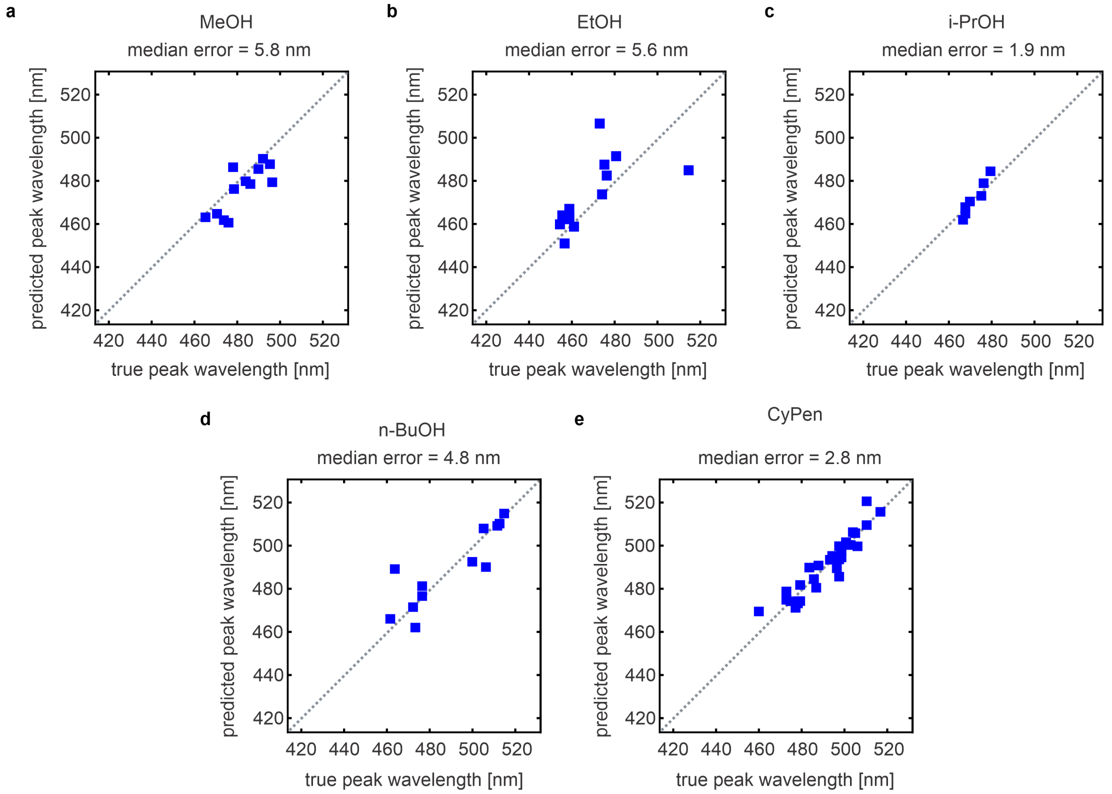

Supporting Figure 6: Prediction accuracy of PL peak wavelength for S-type data. LOO regression plots for PL peak wavelength prediction in syntheses with different antisolvents, shown for a) MeOH, b) EtOH, c) i-PrOH, d) n-BuOH and e) CyPen. A comparison between true PL peak wavelength and predicted PL peak wavelength yields low median errors ($< \pm$ 6 nm) and confirms a high prediction accuracy for all antisolvents. The mean errors are 6.7 nm (MeOH), 9.0 nm (EtOH), 2.6 nm (i-PrOH), 6.4 nm (n-BuOH) and 3.5 nm (CyPen).

4 Optimization of Full Width at Half Maximum

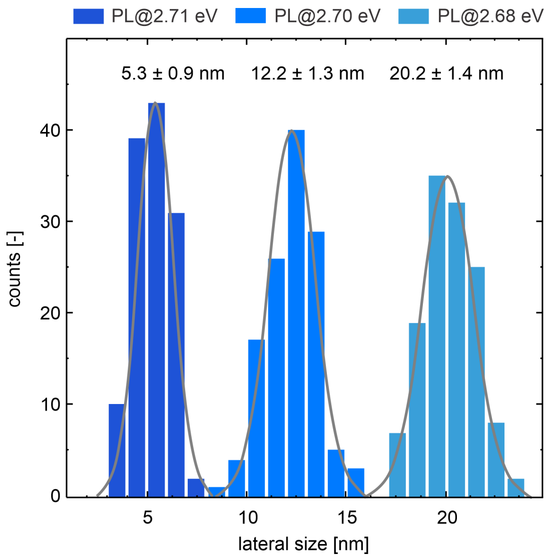

Supporting Figure 7: Lateral size distribution of 3 monolayer (ML) $CsPbBr_3$ NPLs. Lateral size distribution of three different 3ML NPL samples with slightly offset PL peak energies at 2.71 eV, 2.70 eV and 2.68 eV. The lateral sizes of NPLs in each sample were determined from TEM images, and amount to (5.3±0.9) nm, (12.2±1.3) nm and (20.2±1.4) nm.

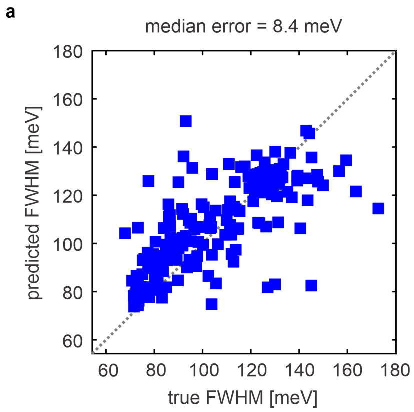

Supporting Figure 8: Prediction accuracy of full width half maximum (FWHM). LOO plots for FWHM predictions of a) the entire dataset with a median error of ± 8.4 meV and a mean error of ± 12.2 meV. Due to the correlation between FWHM and peak energy, there is a chance that the model draws information about the FWHM values from an estimated peak energy as opposed to only modeling the mechanisms that lead to ideal line widths. b) To address this concern, samples with PL peak energy between 2.67 eV and 2.72 eV (3ML NPLs) are shown in a second LOO regression plot. The wide range of predicted FWHM values for this narrow energy interval demonstrates true predictive capabilities of the model. Yet the inherent noise observed in FWHM data might impede reliable predictions and motivates the presented hybrid approach of Gaussian Process regression combined with chemical and physical insights for FWHM optimization. For FWHM predictions, emissive and monodisperse P-type samples were used for training.

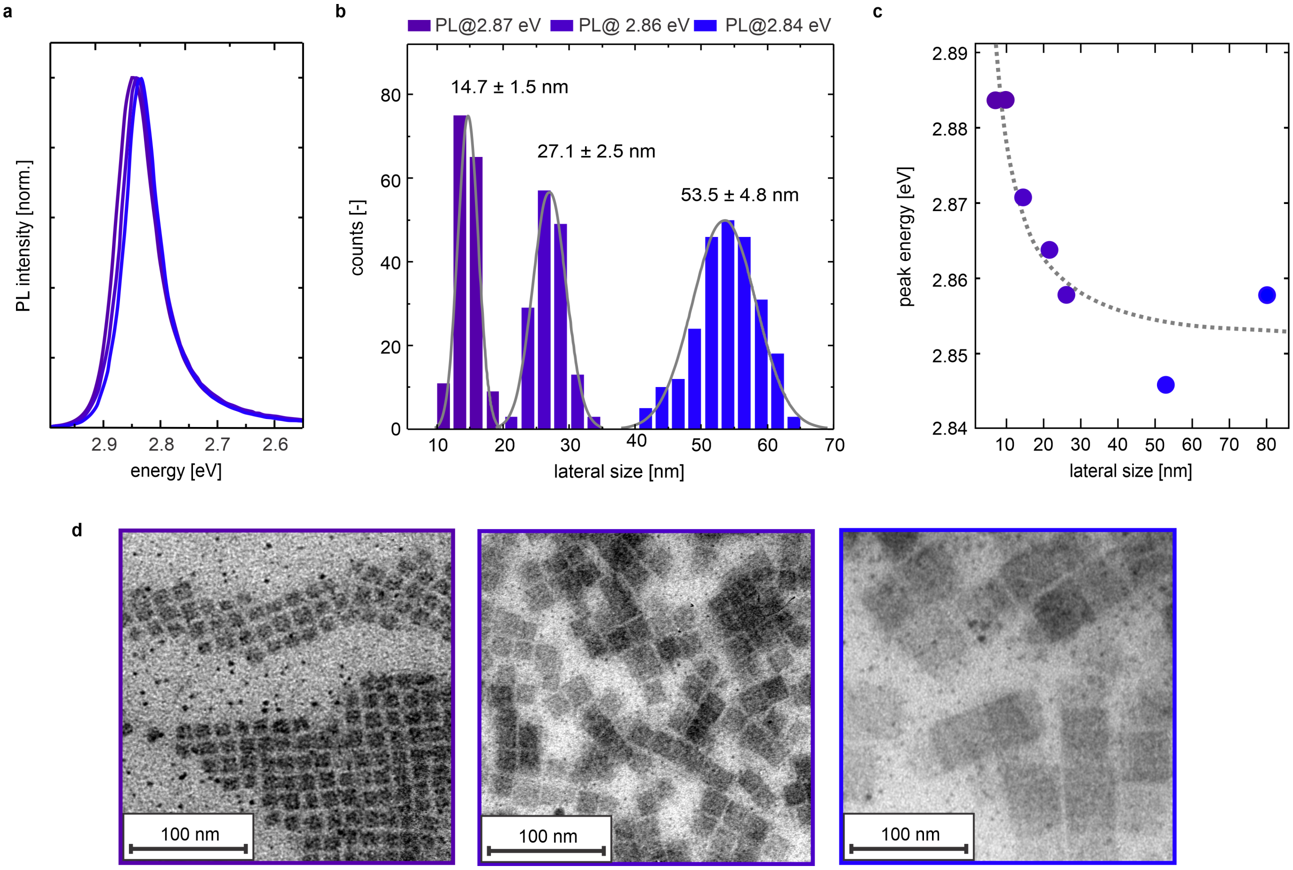

Supporting Figure 9: Lateral confinement tuning in 2ML $CsPbBr_3$ NPLs. a) PL spectra and corresponding b) lateral size distribution of three different 2ML NPL samples with slightly offset PL peak energies at 2.87 eV, 2.86 eV and 2.84 eV. The lateral sizes amount to (14.7±1.5) nm, (27.1±2.5) nm and (53.5±4.8) nm and were determined from corresponding TEM images, shown in d). The correlation between NPL lateral size and PL peak energy, i.e. bandgap energy is adequately described by a power law fit in c). We note that scattering was observed in colloidal solutions of 2ML NPLs with lateral dimensions > 30 nm, most likely due to face-to-face stacking of the NPLs. This can result in reabsorption processes and minor shifts in the PL peak energy.

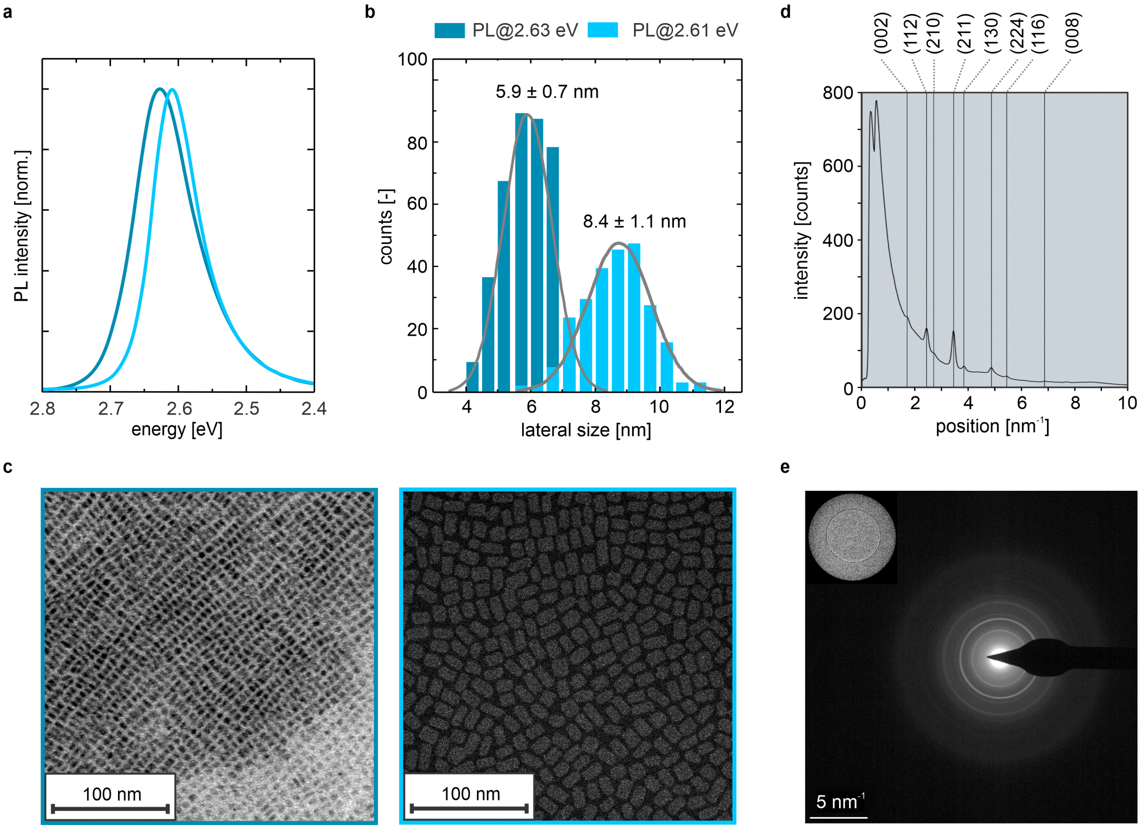

Supporting Figure 10: Lateral confinement tuning in 4ML $CsPbBr_3$ NPLs. a) PL spectra and corresponding b) lateral size distribution of two different 4ML NPL samples with slightly offset PL peak energies at 2.63 eV and 2.61 eV. The lateral sizes amount to (5.9±0.7) nm and (8.4±1.1) nm for the smallest lateral dimensions of NPLs and were determined from corresponding c) TEM and annular dark field scanning transmission electron microscopy (ADF-STEM) images. d) Intensity profile of the diffraction pattern shown in e). Indices were assigned for orthorhombic $CsPbBr_3$. ADF-STEM images of larger 4ML $CsPbBr_3$ NPLs were recorded using a probe-corrected FEI Titan Themis 60-300 operated at an acceleration voltage of 300 kV. Specimen preparation was carried out by dropcasting onto TEM grids (Quantifoil R2/2, 2 nm ultrathin carbon).

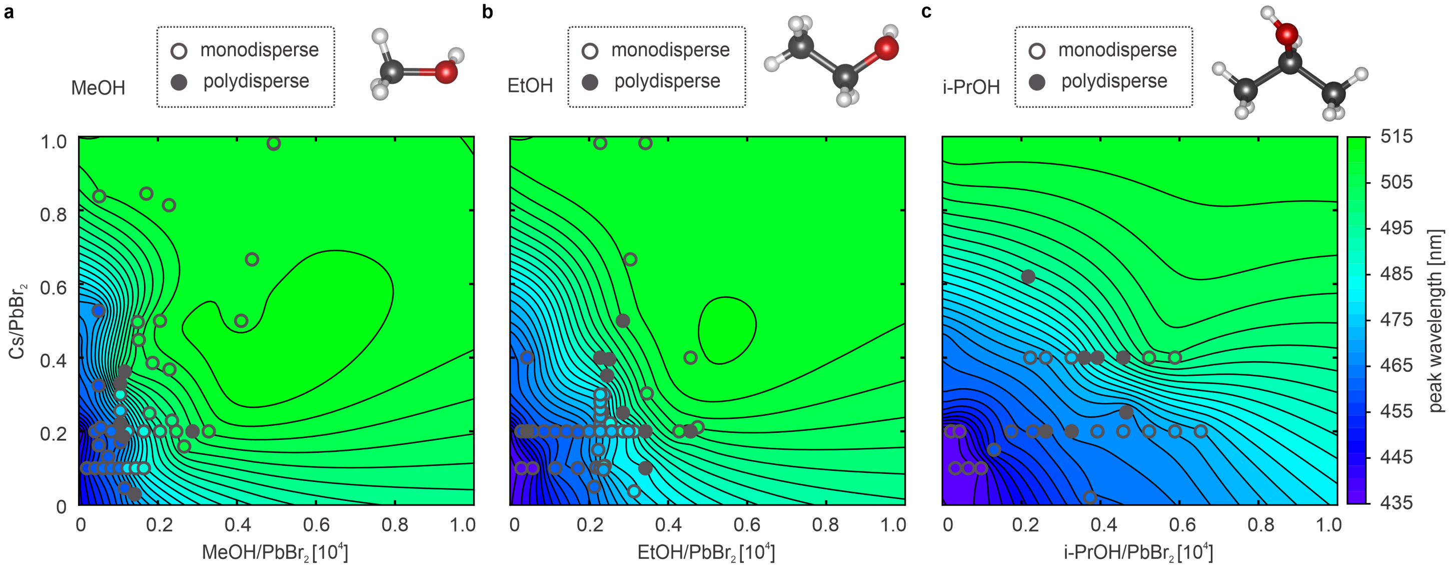

Supporting Figure 11: Two-dimensional representation of parameter space and PL peak wavelength. Contour plots for nanocrystal syntheses with a) MeOH, b) EtOH and c) i-PrOH, showing the characteristic behavior of the PL peak wavelength depending on $Cs/PbBr_2$ and antisolvent/$PbBr_2$ ratio as well as the distribution of monodisperse and polydisperse products in the parameter space. Polydisperse samples with FWHM > 120 meV are more frequently obtained in regions with a high PL peak wavelength gradient.

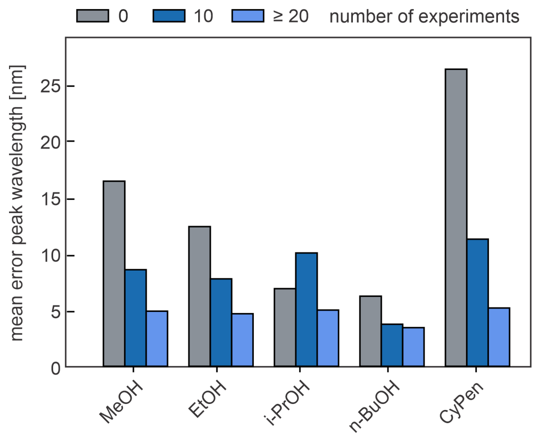

Supporting Figure 12: Mean transfer error of PL peak wavelength prediction for all antisolvents. Basic molecular transfer gives a rough estimation of the PL peak wavelength for unknown antisolvents (0 experiments) with an accuracy strongly dependent on overall geometric similarity. However, performing 10-20 syntheses is sufficient to reach a prediction accuracy of $< \pm 6$ nm (mean error).

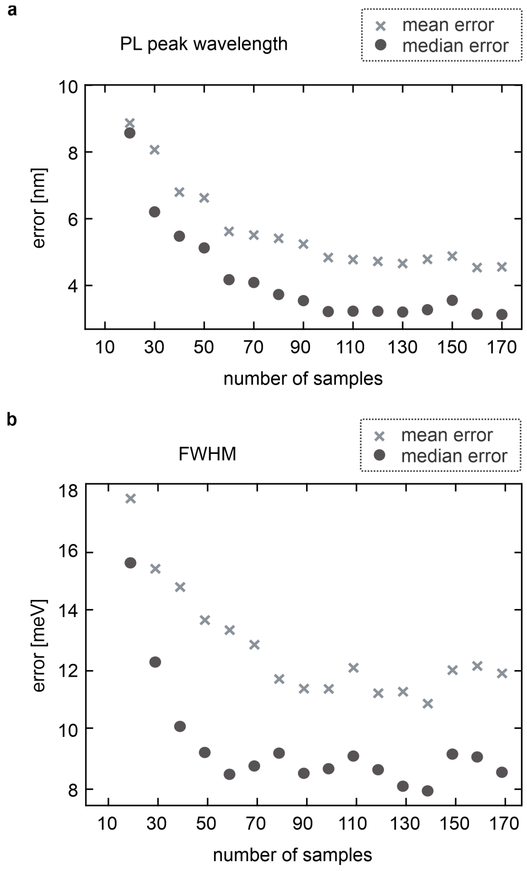

Supporting Figure 13: Evolution of the model performance based on the size of the data set. Starting from an initial 20 data points, at each step, 10 additional samples are added, and a LOO regression accuracy is determined. The evolution of both the mean and median errors is shown for a) the PL peak wavelength prediction and b) the FWHM prediction. Both show a substantial initial improvement for the first 50 data points and a saturation above 100 samples. PLQY is omitted here as it was not possible to produce a reliable prediction in this case.

Supporting Table 5: Solvent properties of tested antisolvents MeOH, EtOH, i-PrOH, n-BuOH and CyPen. Dipole moment μ, Hansen solubility parameters δ_D, δ_P, δ_H and Kamlet-Taft parameters α, β and $\pi^\star$ for all tested (anti)solvents, [1–3] representing the capability of each antisolvent molecule for hydrogen-bonding, dipolar and dispersive interactions, i.e. with precursor monomers or ligand-passivated $CsPbBr_3$ nanocrystals.

antisolvent	μ [D]	δ_D [MPa$^{1/2}$]	δ_P [MPa$^{1/2}$]	δ_H [MPa$^{1/2}$]	α [-]	β [-]	$\pi^\star$ [-]
MeOH	1.7	14.7	12.3	22.3	0.98	0.66	0.60
EtOH	1.7	15.8	8.8	19.4	0.86	0.75	0.44
i-PrOH	1.6	15.8	6.1	16.4	0.76	0.84	0.78
n-BuOH	1.7	16.0	5.7	15.8	0.84	0.84	0.47
CyPen	3.3	17.9	11.9	5.2	0.00	0.52	0.76
toluene	0.4	18.0	1.4	2.0	0.00	0.11	0.54

5 Transmission Electron Microscopy

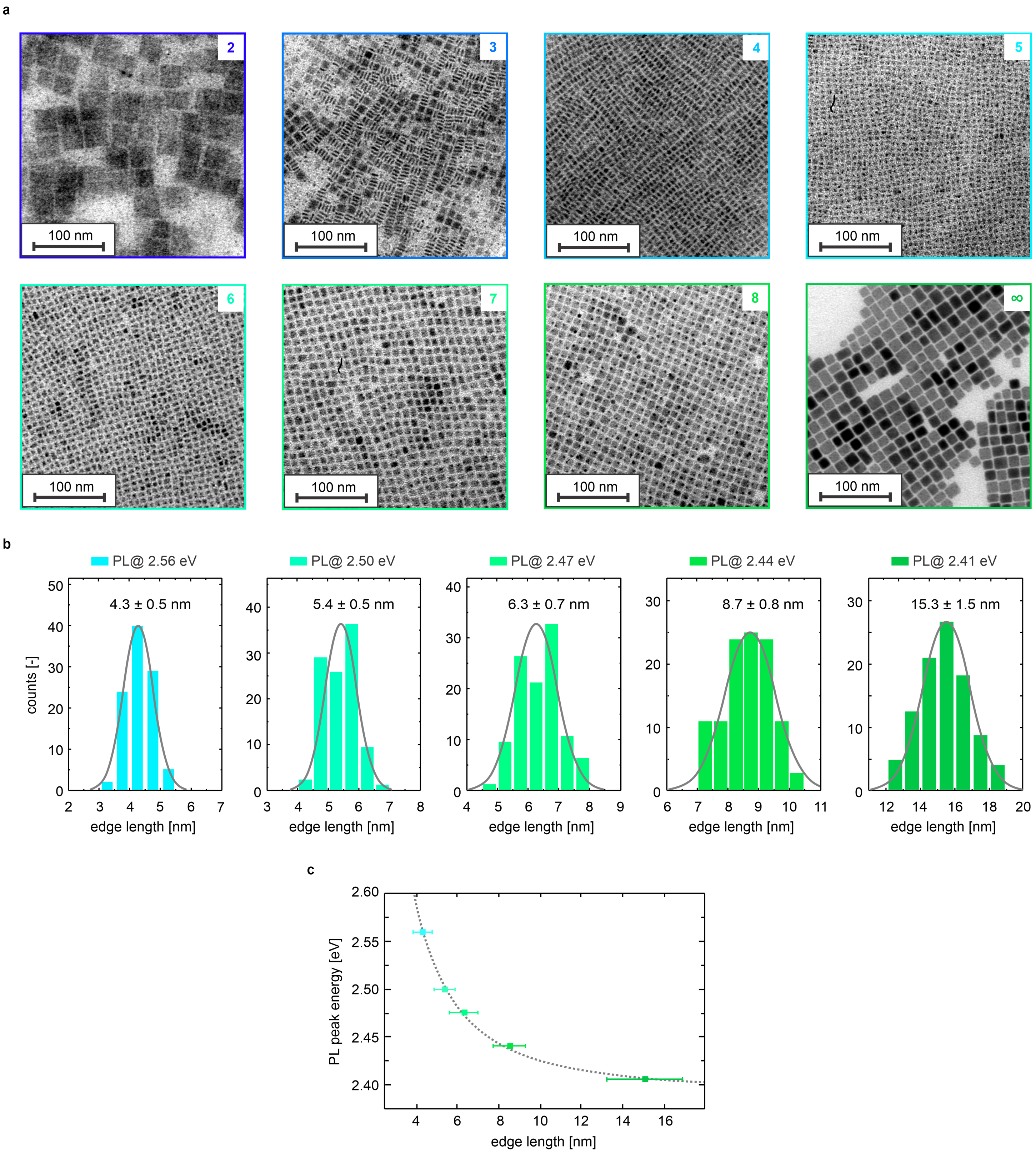

Supporting Figure 14: Morphology of $CsPbBr_3$ nanocrystal products. a) Transmission electron microscopy imaging of various perovskite nanocrystal products obtained by antisolvent-assisted spontaneous crystallization in non-polar solvents. Anisotropic 2ML NPLs, 3ML NPLs and 4ML NPLs (products 2-4) as well as more isotropic, cube-shaped nanocrystals of different sizes (products 5-∞) are shown. b) Size distribution of products 5-∞, all confirming excellent size uniformity of $CsPbBr_3$ nanocrystals. c) The PL peak energy of cube-shaped $CsPbBr_3$ nanocrystals is inversely correlated with the nanocrystal edge length observed in TEM images and can be accurately described with an inverse power law function.

6 Small-Angle X-Ray Scattering

SAXS data was recorded using a Mo K_α microfocus source ($\lambda = 0.71$ Å).[4] The detector was a Pilatus 100K (Dectris) at a sample-detector-distance of 0.808 m and the detector position was calibrated with silver behenate (AgBH) by using pyFAI.[5] SAXS measurements of colloidal $CsPbBr_3$ nanocrystals dispersed in n-hexane (20 mg/mL) were performed in a custom-built cell equipped with a quartz capillary. SAXS data was corrected by subtraction of the instrumental signal, air signal as well as the glass and solvent (n-hexane) signal. A python script with the module jscatter was used for fitting of the SAXS profiles.[6] The nanocrystal sizes determined from TEM images were used as a reference for parameter initialization. Different fit models for cuboid nanocrystals with parameters for edge lengths a, b and c and respective standard deviations were tested. From these, only as many parameters as needed were used to obtain a good fit for the experimental data; two for a true cube (where all edge lengths are identical, $a = b = c$), four for a platelet or rod (two identical edge lengths, $a \neq b = c$) and six for generic cuboid ($a \neq b \neq c$).

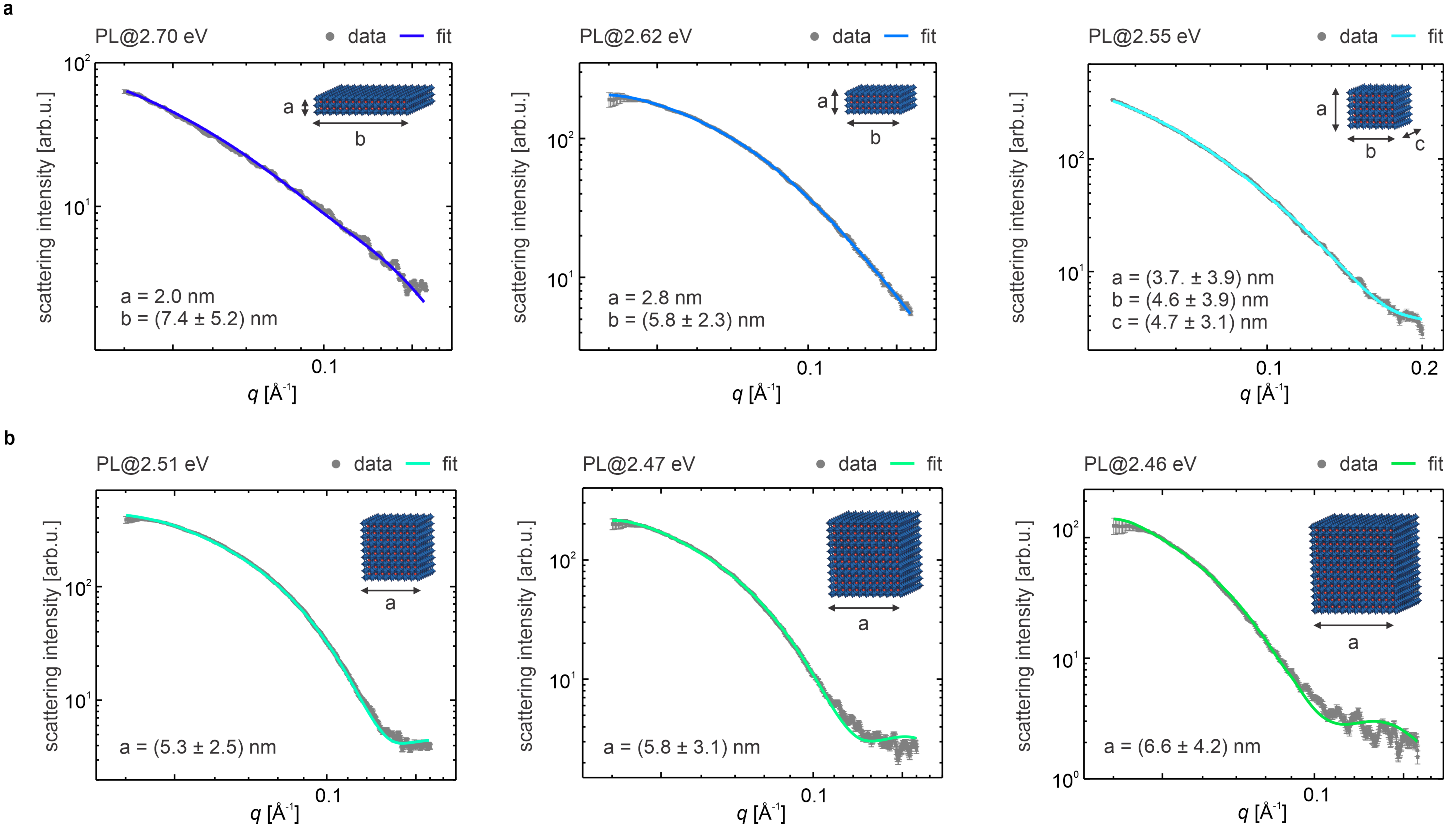

Supporting Figure 15: Small-angle X-ray scattering analysis of $CsPbBr_3$ nanocrystals measured with a laboratory setup. a) The small-angle X-ray scattering (SAXS) intensity of pristine nanocrystals in n-hexane is described very well by a plate model fit (a < b = c) for nanocrystal products 3 and 4. For nanocrystal product 5, a plate model fit of the experimental SAXS data yields two similar values for a and b, hinting at a very minor anisotropy of the perovskite nanocrystals. b) The SAXS intensity of nanocrystal products 6-8 is better described with a cuboid model (a = b = c). These results are in good agreement with the sizes of similar nanocrystal samples observed in TEM images. Therefore, the SAXS anaylsis also confirms the different shape of nanocrystal products 2-4 (NPLs) in contrast to nanocrystal products 5-∞ (nanocubes).

7 Photoluminescence Quantum Yield

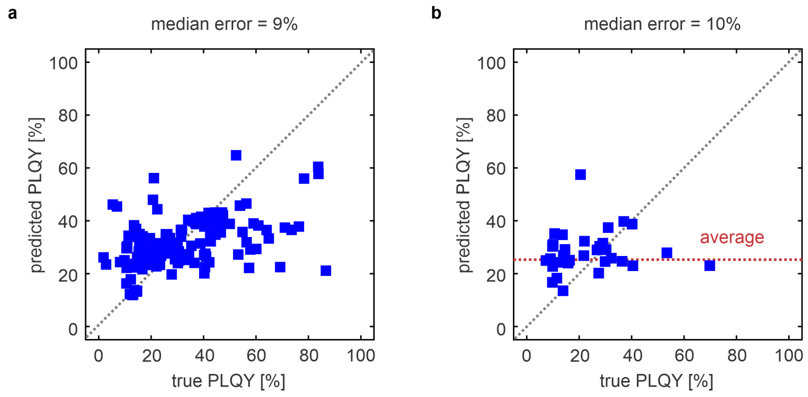

Supporting Figure 16: Prediction accuracy of photoluminescence quantum yield (PLQY). LOO plots for PLQY predictions of a) the entire dataset, for which the trained model has some predictive power, and b) samples with PL peak energies between 2.67 eV and 2.72 eV (3ML NPLs), for which the trained model predicts an average PLQY value. This behavior is a direct consequence of the correlation between nanocrystal product and PLQY. Trained on all data points, the model accurately predicts the PL peak wavelength and estimates the PLQY based on this quantity. However, this approach fails for a narrower emission range, i.e. for a single type of NC product. Any further correlation between synthesis parameters and the observed PLQY is likely overshadowed by the inherent noise with regards to this target. For PLQY predictions, monodisperse S-type and P-type data were used for training.

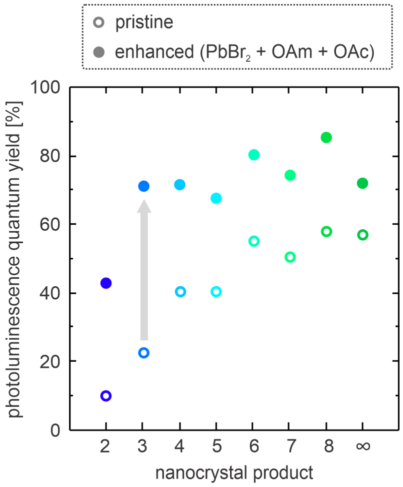

Supporting Figure 17: Photoluminescence quantum yield of pristine and enhanced $CsPbBr_3$ nanocrystals. Comparison of PLQY values for pristine perovskite nanocrystals as obtained from antisolvent-assisted synthesis in ambient conditions and those treated with 5-10% volume of enhancement solution containing $PbBr_2$, OAm and OAc in n-hexane. The post-synthesis treatment passivates surface vacancies and notably improves PLQY values of all $CsPbBr_3$ nanocrystal products, up to 80-90%.

8 $CsPbI_3$ Nanocrystals

Supporting Table 6: Classification of $CsPbI_3$ nanocrystals. Perovskite nanocrystal were sorted into different groups according to the PL peak wavelength or PL peak energy. The upper and lower limit for each nanocrystal product is listed.

	lower limit [nm]	upper limit [nm]	upper limit [eV]	lower limit [eV]
3	580	610	2.14	2.03
4	611	635	2.03	1.95
5	640	680	1.94	1.82
∞	681	700	1.82	1.77

Supporting Table 7: Optimized synthesis parameters for each $CsPbI_3$ nanocrystal product. Precursor concentrations and volumes, as well as resulting Cs/PbI_2 ratio for the synthesis of optimized nanocrystal products with the narrowest PL profiles.

nanocrystal product	V_{PbI_2} [µL]	c_{PbI_2} [M]	V_{Cs} [µL]	c_{Cs} [M]	Cs/PbI_2	FWHM [meV]
3	4000	0.01	10	0.2	0.05	86
4	2000	0.01	50	0.2	0.50	89
5	2000	0.01	80	0.2	0.80	139
∞	1000	0.01	100	0.1	1.00	82

References

[1] D. R. Lide, *CRC Handbook of Chemistry and Physics, 84th Edition*, CRC Press, **2003**, ISBN 0849304849.

[2] C. M. Hansen, *Hansen Solubility Parameters: A User's Handbook, Second Edition*, CRC Press, **2007**, ISBN 9780429127526.

[3] M. J. Kamlet, J. L. M. Abboud, M. H. Abraham, R. W. Taft, Linear Solvation Energy Relationships. 23. A Comprehensive Collection of the Solvatochromic Parameters, pi*, alpha, and beta, and Some Methods for Simplifying the Generalized Solvatochromic Equation, *The Journal of Organic Chemistry* **1983**, *48*, 17 2877–2887, https://doi.org/10.1021/jo00165a018.

[4] L. Bruetzel, S. Fischer, A. Salditt, S. Sedlak, B. Nickel, J. Lipfert, A Mo-Anode-based in-house Source for Small-Angle X-ray Scattering Measurements of Biological Macromolecules, *Rev. Sci. Instrum.* **2016**, *87*, 2, https://doi.org/10.1063/1.4940936.

[5] J. Kieffer, D. Karkoulis, PyFAI, a Versatile Library for Azimuthal Regrouping, *Journal of Physics: Conference Series* **2013**, *425*, 20 202012, https://doi.org/10.1088/1742-6596/425/20/202012.

[6] R. Biehl, Jscatter, a Program for Evaluation and Analysis of Experimental Data, *PLOS ONE* **2019**, *14*, 6 e0218789, https://doi.org/10.1371/journal.pone.0218789.
